# Comparative proteomic analysis of *Ulva prolifera* response to high temperature stress

**DOI:** 10.1186/s12953-018-0145-5

**Published:** 2018-10-27

**Authors:** Meihua Fan, Xue Sun, Zhi Liao, Jianxin Wang, Yahe Li, Nianjun Xu

**Affiliations:** 1 Marine Sciences and Technology College, Zhejiang Ocean University, Zhoushan, Zhejiang, 316000 China; 2 Key Laboratory of Marine Biotechnology of Zhejiang Province, School of Marine Sciences, Ningbo University, Ningbo, Zhejiang, 315211 China

**Keywords:** *Ulva prolifera*, Proteome analysis, High temperature, iTRAQ

## Abstract

**Background:**

*Ulva prolifera* belongs to green macroalgae and is the dominant species of green tide. It is distributed worldwide and is therefore subject to high-temperature stress during the growth process. However, the adaptation mechanisms of the response of *U. prolifera* to high temperatures have not been clearly investigated yet.

**Methods:**

In this study, isobaric tags for relative and absolute quantitation (iTRAQ) labelling was applied in combination with the liquid chromatography-tandem mass spectrometry (LC-MS/MS) to conduct comparative proteomic analysis of the response of *U. prolifera* to high-temperature stress and to elucidate the involvement of this response in adaptation mechanisms. Differentially expressed proteins (DEPs) of *U. prolifera* under high temperature (denote UpHT) compared with the control (UpC) were identified. Bioinformatic analyses including GO analysis, pathway analysis, and pathway enrichment analysis was performed to analyse the key metabolic pathways that underlie the thermal tolerance mechanism through protein networks. Quantitative real-time PCR and western blot were performed to validate selected proteins.

**Results:**

In the present study, 1223 DEPs were identified under high temperature compared with the control, which included 790 up-regulated and 433 down-regulated proteins. The high-temperature stimulus mainly induced the expression of glutathione S-transferase, heat shock protein, ascorbate peroxidase, manganese superoxide dismutase, ubiquitin-related protein, lhcSR, rubisco activase, serine/threonine protein kinase 2, adenylate kinase, Ca^2+^-dependent protein kinase (CDPK), disease resistance protein EDS1, metacaspase type II, NDPK2a, 26S proteasome regulatory subunit, ubiquinone oxidoreductase, ATP synthase subunit, SnRK2s, and cytochrome P450. The down-regulated proteins were photosynthesis-related proteins, glutathione reductase, catalase-peroxidase, thioredoxin, thioredoxin peroxidase, PP2C, and carbon fixation-related proteins. Furthermore, biological index analysis indicated that protein content and SOD activity decreased; the value of Fv/Fm dropped to the lowest point after culture for 96 h. However, APX activity and MDA content increased under high temperature.

**Conclusion:**

The present study implied an increase in proteins that were associated with the stress response, oxidative phosphorylation, the cytokinin signal transduction pathway, the abscisic acid signal transduction pathway, and the glutathione metabolism pathway. Proteins that were associated with photosynthesis, carbon fixation in photosynthesis organisms, and the photosynthesis antenna protein pathway were decreased. These pathways played a pivotal role in high temperature regulation. These novel proteins provide a good starting point for further research into their functions using genetic or other approaches. These findings significantly improve the understanding of the molecular mechanisms involved in the tolerance of algae to high-temperature stress.

**Electronic supplementary material:**

The online version of this article (10.1186/s12953-018-0145-5) contains supplementary material, which is available to authorized users.

## Background

*Ulva prolifera* (Chlorophyta) belongs to Chlorophyta, Ulvales, Ulvaceae and is a type of adaptable large algae. It is rich in protein, carbohydrates, crude fibre, vitamins, amino acids, unsaturated fatty acids and other bioactive substances. *U*. *prolifera* was developed as a food material, for refining raw materials, and as aquaculture feed. The natural reproductive ability of *U. prolifera* is strong, and it is the dominant species in the Yellow Sea and East China Sea. The breakout of *U. prolifera* in Qingdao since June 2008 has destroyed the natural environment and has a great influence on tourism and the aquatic industry. In addition, *U. prolifera* had been abundant in Xiangshan of the East China Sea from January to March. However, it disappeared in April. Therefore, change in the environment, especially temperature, had a great influence on the growth of *U. prolifera.* To adapt to these environmental changes, *U. prolifera* has developed mechanisms to adapt to different types of stresses including high temperatures, cold, hypersalinity and ultraviolet radiation [1, 2]. High temperature is a major environmental factor that limits *U. prolifera* yield. It can affect photosynthesis, respiration, water balance, membrane stability, hormone levels, and primary and secondary metabolites [3]. Direct injuries that are associated with high temperature include protein denaturation, aggregation, and increased fluidity of membrane lipids. Indirect or slower heat injuries include inactivation of enzymes in chloroplasts and mitochondria, inhibition of protein synthesis, protein degradation and loss of membrane integrity [4–6]. Photosynthesis is a process that is very sensitive to heat stress. The inhibition of photosystem II (PSII) leads to an invariable change in chlorophyll fluorescence [7]. To counter the effects of heat stress on cellular metabolism, plants respond to temperature changes by reprogramming their transcriptome, proteome, metabolome and lipidome. Such changes are aimed at establishing a new steady-state balance of metabolic processes that can enable the organism to function, survive and even reproduce at a higher temperature [5]. Previous studies have focused on physiological changes including photosynthesis, respiration, cell-membrane stability, hormone changes and induced antioxidant systems and heat-shock-protein expression in *U. prolifera* at high temperatures [8–10]. The effect of heat stress on *U. prolifera* appears to be associated with multiple processes and mechanisms including stress-related genes, transcription factors, and metabolism [11]. However, the transcription patterns are not always directly consistent with protein expression levels. To the best of our knowledge, the transcriptome expression analysis of the responses of *U. prolifera* to abiotic stresses such as low light or low temperature have been reported [1]. However, little attention has been given to the proteomic changed analysis of the response of *U. prolifera* to high temperatures.

Recent advances in the use of iTRAQ labelling of peptides and proteins for the relative quantification of an entire organism or cell proteome under different experimental conditions offer promising tools for the discovery of biomarkers at the protein level. Proteomic responses to heat stress have been widely studied in many species, such as *Aspergillus flavus* [12], *Phaeodactylum tricornutum* [13], and *Pyropia yezoensis* [14]. In addition, proteomic analysis also has been used to identify the metabolic responses of *Synechocystis* PCC 6803 to biofuel butanol, ethanol and hexane treatments [15–17]. However, it has been rarely reported in *U. prolifera.*

We describe here a procedure for proteomic analysis of the *U. prolifera* using iTRAQ labelling to identify differentially expressed proteins upon exposure to high temperature. The goal of the study is to use the molecular information in *U. prolifera* and provide a better understanding of the thermotolerance mechanism in *U. prolifera* and other macroalgae of the same habitats*.*

## Methods

### Macroalgae collection and preparation

Macroalgae used for this study were collected from the intertidal zone of the Xiangshan port of the East China Sea, Ningbo, China (121.82424 E, 29.552086 N) during low tide. *U. prolifera* samples were extensively rinsed with filtered (0.2 μm) seawater and placed into an enamel tray to further remove debris and epiphytes. Furthermore, the *U. prolifera* samples were disinfected with 0.2% KI for 10 min and flushed with sterile seawater. Next, *U. prolifera* samples that were inoculated in Provasoli medium [18] with 200 μg/mL ampicillin were placed in a biochemical incubator that was set to the appropriate temperature (25 °C) and light intensity (100 μmol s^− 1^ m^− 2^ on a 12 h: 12 h light dark cycle). To obtain relatively sterile materials, the *U. prolifera* samples were disinfected and transferred to fresh Provasoli medium every 7 days and continuously cultured for 10 days at 25 °C.

### Experimental design

*U. prolifera* samples were transferred to a high-temperature 35 °C growth chamber and subjected to the same irradiance and photoperiod (denoted UpHT) as the treatment group, with three biological repeats. In addition, the *U. prolifera* samples were cultured at 25 °C as the control group (denoted UpC), with three biological repeats. The gametophyte thalli were harvested at 3 h, snap frozen using liquid nitrogen and maintained at − 70 °C until protein extraction. In addition, *U. prolifera* were collected at 3 h, 12 h, 24 h, 48 h and 96 h for the determination of physiological indexes.

### Protein extraction

Proteins were extracted using the following method: the *U. prolifera* samples were disrupted in lysis buffer (including 0.1 M/L Tris-HCl, 1.4 M/L NaCl, 0.02 M/L Na_2_EDTA, 2% CTAB, 0.1% DIECA, 2% PVP K-30, 0.2% β-mercaptoethanol with pH adjusted to 8.0) with enzyme inhibitors (phenylmethyl sulphonyl fluoride, PMSF) using a tissue grinder and were sonicated on ice. The expected proteins were extracted after centrifugation at 25,000 g for 20 mins. The supernatants were carefully removed and mixed with an appropriate volume of cold acetone and stored at − 20 °C overnight. The mixture was centrifuged again. The pellets were dissolved using lysis buffer. Next, 10 mM DTT (dithiothreitol) was added to the solution, and the solution was maintained at 56 °C for 1 h to reduce the disulphide bonds of peptides. Next, 55 mM IAM (iodacetamide) was added to the solution, and the solution was maintained in a darkroom for 45 min. An appropriate volume of chilled acetone was added into the solution, which was maintained at − 20 °C for 2 h. The pellet was dissolved with lysis buffer to obtain the protein solution. Final protein concentrations were determined using BCA kits according to the manufacturer’s instructions.

### Protein digestion and iTRAQ labelling

Each 100 μg of protein was digested in trypsin solution (1:10) and incubated at 37 °C for 4 h. The digested peptides were labelled using iTRAQ reagents according to the manufacturer’s instructions. iTRAQ Reagent 6-Plex kit (Applied Biosystems, Foster City, CA, USA) was used for iTRAQ labelling according to the manufacturer’s protocol with some modifications. The iTRAQ labelling reagents 113, 114 and 115 were used to label three biological replicates from the control group; similarly, 116, 117 and 118 were used to label three biological replicates from UpHT.

### Peptide fractionation via reverse phase chromatography

Prominence high-performance liquid chromatography (HPLC) system (LC-20AB; Shimadzu, Kyoto, Japan) that was connected to a reversed-phase column was used to perform the first dimension of peptide separation. The iTRAQ-labelled sample was dried using a speedvac and resuspended in 2 mL of 5% acetonitrile, and the pH was adjusted to approximately pH 9.8. The resuspended sample solution was injected into a Gemini C18 column (4.6 × 250 mm, 5-μm particles). The following separation gradient with buffer B (98% ACN, 0.1% FA) was used at a flow rate of 1000 μL/min: 5% of mobile phase B in 10 min, 5 to 35% of mobile phase B in 40 min, and 35 to 95% of mobile phase B in 1 min. The system was then maintained in 95% of mobile phase B in 3 min, which was decreased to 5% within 1 min before equilibrating with 5% solvent B for 10 min. Elution was monitored by measuring the absorbance at 214 nm, and fractions are collected every 1 min. The peptides were subjected to nanoelectrospray ionization followed by tandem mass spectrometry (MS/MS) in a Q EXACTIVE (Thermo Fisher Scientific, San Jose, CA) that was coupled online to the HPLC.

### Identification and quantification of peptides and proteins

The MS/MS data were searched against a protein sequence database that was derived from the transcriptome database of *U. prolifera* and partion for other green algae proteins using the ProteinPilot™ software 4.2 (SCIEX) for peptide identification and quantification. The MS/MS spectra obtained were searched using the following user-defined search parameters. The MS/MS spectra were searched against a decoy database to estimate the false discovery rate (FDR) for peptide identification. For iTRAQ studies, 95% protein confidence level was used as the identification criterion.

### Proteomics data processing

The raw MS/MS data were converted into “.mgf” files using ProteinPilot software (AB Sciex). Mascot version 2.3.0 (Matrix Sciences, London, UK) was used to search against the transcriptome database of *U. prolifera* and partion for other green algae proteins. To identify false positives, raw spectra from the actual database were compared with a generated database of random sequences. Only peptides with significant scores at the 95% confidence level were considered reliable and used for protein identification. Protein quantitative ratios were weighted and normalized relative to the median ratio in Mascot. Only proteins with significant quantitative ratios between the two treatments (*p* < 0.05) and with fold changes > 1.2 or < 0.83 were considered differentially expressed. Functional annotation and classification of proteins for the DEPs was conducted using the Blast2GO program (https://www.blast2go.com/) [19]. KEGG pathway analysis was performed using (http://kobas.cbi.pku.edu.cn/anno_iden.php). The clustering of the heat map was conducted using Mev4.9. Enrichment analysis was conducted using the singular enrichment analysis (SEA) tool in the agriGO toolkit [20]. Subcellular localizations of proteins were determined using TargetP [21].

### RNA isolation and qRT-PCR

The isolated RNA sequencing samples were also used to perform real-time quantitative (qRT-PCR) analysis. Extraction of total RNA from three biological replicates in UpHT and three biological replicates in UpC was performed using plant RNA Kit (Omega). The total RNA was reverse transcribed to cDNA using TransScript® All-in-One First-Strand cDNA Synthesis SuperMix for qPCR (one-step gDNA removal) (Trans) according to the manufacturer’s protocol. The purity and integrity of the RNA was verified via absorbance measurements at 260 nm using a Nanodrop ND-1000 spectrophotometer (NanoDrop Technologies Inc., USA) and via electrophoretic separation using a Bioanalyzer and the RNA Nano kit (Agilent Technologies). From the DEGs, 8 related genes were selected to verify the reliability of the transcriptome by real-time fluorescence quantitative PCR. Quantitative real-time PCR was performed on an ABI Quant Studio6 Flex (USA) using SYBR Green PCR kits (Trans, China) according to the manufacturers’ instructions. Each cycle of the thermal amplification followed the universal protocol according to the manufacturer’s instructions: 94 °C for 30 s, followed by 40 cycles with 15 s at 94 °C and 1 min at 55 °C. For all qRT-PCR results, β-actin was used as the housekeeping gene, for which sequences of the specific primers have been previously published. The gene-specific primers used to amplify each gene were as follows: β-actin forward primer was 5’-AGGATGCATACGTTGGTGAA-3’, and β-Actin reverse primer was 5’-TTGTGGTGCCAAATCTTCTC-3’ [8].

#### Western blot

The protein samples were electrophoresed via 12% SDS-PAGE and transferred onto nitrocellulose membranes (Millipore, Germany). After blocking with 5% skim milk, the membranes were incubated with the rabbit polyclonal anti-HSP70 (Agrisera AS0837, Sweden,1: 3000), Rabbit polyclonal Rubisco large subunit,form I and form II antibody (Agrisera AS03037, Sweden 1:10000) at 4 °C overnight, respectively. Subsequently, the membranes were washed with PBS five times and incubated with goat anti-rabbit IgG(H + L) secondary antibody (Thermo Fisher Scientific, USA) at a ratio of 1:5000 at room temperature for 2 h. Finally, the membranes were developed using an enhanced chemiluminescence kit (Santa Cruz, USA^)^ [22].

#### PPI network analysis

The PPI data of *U. prolifera* were downloaded from the STRING database. Each interaction has a combined score, which represents the reliability of the interaction between the proteins. The PPI interactions with a combined score (0: lowest confidence; 1: highest confidence) larger than 0.4 were used for further network analysis. All differentially expressed proteins were mapped onto the PPI network, and the Cytoscape tool was used to visualize the network.

### Physiological and biochemical indicators

It was proposed that physiological adaptations of *U. prolifera* may enable it to survive the harsh intertidal environment and contribute to subsequent blooms. To investigate the effects of high temperature on antioxidant activities and photosynthesis indexes, the *U. prolifera* samples were quickly frozen using liquid nitrogen and stored at − 80 °C. For antioxidative enzyme extraction, 1.0 g of *U. prolifera* was homogenized in 5.0 mL of extraction buffer containing 1 mM EDTA, 0.05% Triton-X-100, 2% PVP, and 1 mM ascorbate in 50 mM phosphate buffer, pH 7.8. This mixture was centrifuged at 12,000 g for 20 min at 4 °C [11, 23]. The resulting supernatant was stored at − 20 °C for the assay of the following antioxidant enzymes.

### Superoxide dismutase activity determination

Total superoxide dismutase activity was determined via the inhibition of the photochemical reduction of the chloride nitroblue tetrazolium (NBT) at 560 nm. The enzyme activity was expressed as unit U/g fresh weight, and one SOD unit was defined as the quantity required to inhibit the photoreduction rate of NBT by 50% [24].

### Ascorbate peroxidase (APX) activity determination

Ascorbate peroxidase (APX) activity was determined by measuring the decrease in absorbance of ascorbic acid (AsA) at 290 nm. The reaction mixture contained 2.6 mL of PBS (pH 7.5, containing 0.1 mmol/L EDTA and 0.5 mmol/L AsA), 0.1 mL of sample, and 0.3 mL of 2 mmol/L H_2_O_2_ [24, 25].

### Estimation of lipid peroxidation (MDA content)

Fresh samples (500 mg each) were homogenized in 10 mL of 0.1% trichloroacetic acid (TCA). The homogenate was centrifuged at 15,000 g for 5 min. Next, 2 mL aliquot of supernatant was taken and 4 mL of 0.5% thiobarbituric acid (TBA) in 20% TCA was added to it. The mixture was heated at 95 °C for 30 min and quickly cooled in an ice bath. After centrifugation at 10,000 g for 10 min to remove the suspended turbidity, the absorbance of the supernatant was recorded at 532 nm absorbance on a UV-visible spectrophotometer (Chemito Spectrascan, UV 2600). The value of non-specific absorption at 600 nm was subtracted. The MDA content was calculated using its absorption coefficient of 155 mmol/ cm [26, 27].

### Chlorophyll fluorescence parameters measurements

Chlorophyll fluorescence parameters of the maximum photochemical efficiency (Fv/Fm) were measured using Water-PAM. Detection of chlorophyll fluorescence was made using a pulse modulation fluorometer (JUNIOR-PAM, Walz, Germany). At least three algal samples were used for each measurement of chlorophyll fluorescence, and the algae were acclimated to darkness for 10 min before being analysed. The maximum quantum yield of the photosystem (PS) II of *U. prolifera* was estimated as *F*V/*Fm*; the photochemical quenching coefficient (qP) and nonphotochemical quenching coefficient (NPQ) were also determined. The rapid light curves (RLCs) consisted of the fluorescence response to eight different and increasing actinic irradiance levels over the range of 0 ∼ 820 μmol photons m^− 2^ s^− 1^. The parameters of the RLCs were calculated following the formula described by Jassby and Platt: rETR (relative electron transport rate) = rETRmax × tan*h*( × *I*/ rETRmax), where rETRmax is the saturated maximum rETR, tan*h* is the hyperbolic tangent function, is the initial slope of the RLC (the efficiency of the electron transport), and *I* is the incident irradiance [28].

## Results and discussion

### Annotation of proteome data

**Fig. 1:**
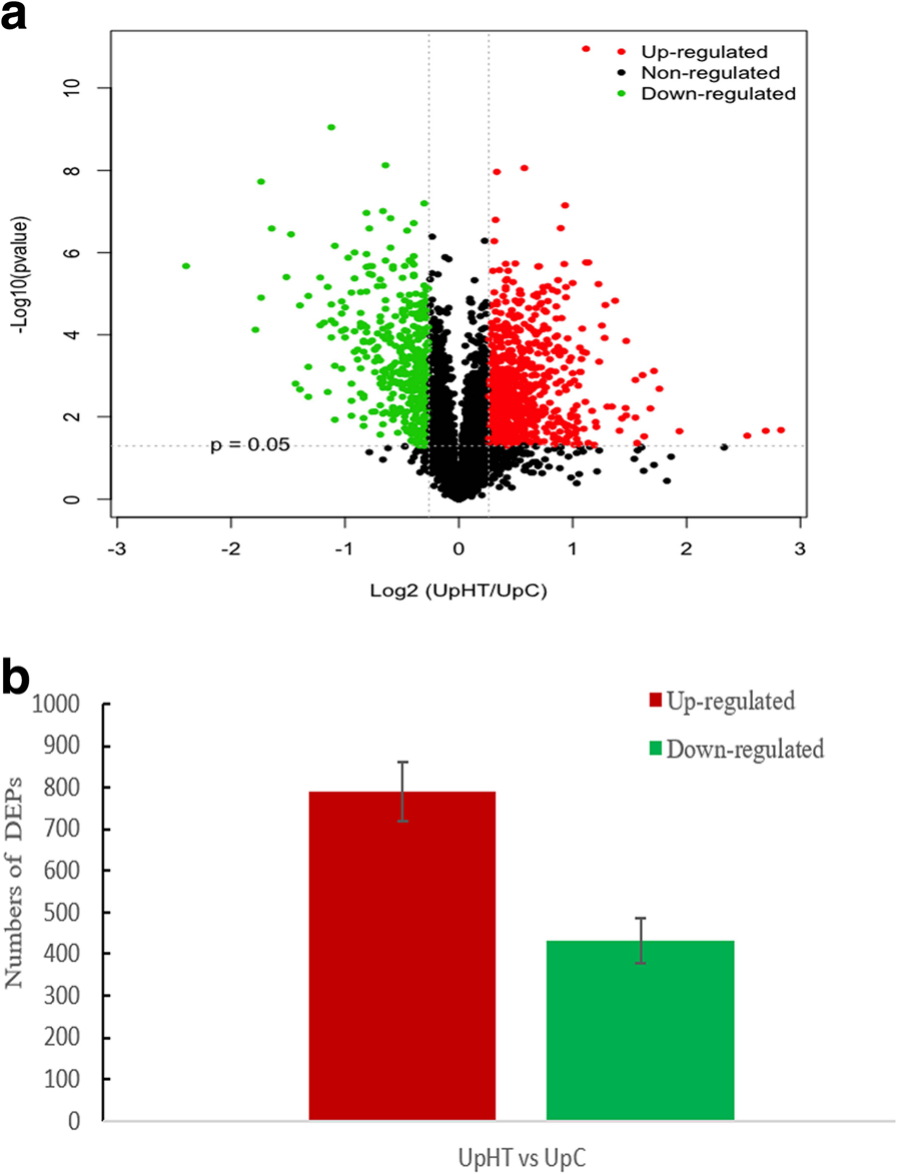
Identification of DEPs. **a** Volcano of differentially expressed proteins(DEPs)under UpHT comparing to UpC. This plot depicts volcano plot of log2 fold-change (x-axis) versus -log10 Qvalue (y-axis, representing the probability that the protein is differentially expressed). Qvalue<=0.05, Foldchange> 1.2 and < 0.83 are set as the significant threshold for differentially expression. Dots in green indicated down-regulated proteins, Dots in red indicated up-regulated proteins, black indicated no significantly change. **b** the number of DEPs (UpHT vs UpC)

Protein response to heat stress in *U. prolifera* were revealed by iTRAQ analysis. A total of 283,344 spectra were generated; 15,546 peptides and 4449 proteins were identified with the cut-off of Mascot Percolator Q value<= 0.01 [29]. Differentially expressed proteins (DEPs) were identified upon an expression ratio > 1.20 or < 0.83 and *p* < 0.05 under UpHT compared to UpC [30–32]. During high-temperature stress, a total of 1223 proteins were found to be significantly changed, of which, 790 DEPs were up-regulated and 433 DEPs were down-regulated (Fig. 1, Table 1), which indicated that almost 27.50% of the identified proteins changed their abundance significantly in response to the heat stress.

**Table 1:** Partion DEPs under UpHT vs UpC conditions

	Protein_ID	Description	AccessionNo.	GO annotation
Up-regulated				
454	CL232_Contig1_All	glutathione S-transferase [*Chlorella variabilis*]	gi|307,109,344|gb|EFN57582.1|	antioxidant activity
1426	Unigene12106_All	glutathione S-transferase [*Coccomyxa subellipsoidea* C-169]	gi|384,253,101|gb|EIE26576.1|	catalytic_activity
1483	CL2198_Contig2_All	glutathione S-transferase [*Coccomyxa subellipsoidea* C-169]	gi|384,253,101|gb|EIE26576.1|	catalytic_activity
695	Unigene6725_All	minus strand polyubiquitin [*Aureococcus anophagefferens*]	gi|323,454,622|gb|EGB10492.1|	antioxidant activity
2681	CL6691_Contig2_All	MnSOD [*U. prolifera*]	gi|149,275,665|gb|ABR23158.1|	antioxidant activity
202	Unigene40313_All	heat shock protein 90, cytosolic [*Ostreococcus lucimarinus* CCE9901]	gi|166,770|gb|AAA32822.1|	binding
984	gi|297,592,060|gb|ADI46845_1|	heat shock protein 70B [*Volvox carteri f_ nagariensis*]	gi|302,853,561|ref.|XP_002958295.1|	binding
2451	CL5666_Contig1_All	heat shock protein 90 [*Ulva pertusa]*	gi|371,940,442|dbj|BAL45643.1|	binding
2543	CL2081_Contig1_All	heat shock protein 90C [*Chlamydomonas reinhardtii*]	gi|159,490,014|ref.|XP_001702984.1|	binding
2944	gi|300,269,068|gb|EFJ53248_1|	heat shock protein Hsp70H [*Volvox carteri* f_ *nagariensis*]	gi|302,829,372|ref.|XP_002946253.1|	binding
3056	gi|158,270,891|gb|EDO96722_1|	heat shock protein 90C [*Chlamydomonas reinhardtii*]	gi|159,490,014|ref.|XP_001702984.1|	binding
3197	gi|254,558,246|gb|ACT67905_1|	heat shock protein 70 [*Ulva pertusa*]	gi|254,558,246|gb|ACT67905.1|	binding
4221	CL413_Contig1_All	minus strand heat shock protein 60 [*Ulva pertusa*]	gi|371,940,440|dbj|BAL45642.1|	binding
75	CL152_Contig5_All	ubiquitin-60S ribosomal protein L40 [*Vitis vinifera*]	gi|359,482,015|ref.|XP_002282071.2|	structural_molecule_activity
2429	Unigene16018_All	NADH:ubiquinone oxidoreductase 11 kDa subunit [*Chlamydomonas reinhardtii*]	gi|159,475,537|ref.|XP_001695875.1|	catalytic_activity
2470	gi|1,001,185,371|gb|AML80572_1|	NADH-ubiquinone oxidoreductase 49 kDa subunit (mitochondrion) [*U. prolifera*]	gi|49,147,198|ref.|YP_025791.1|	binding catalytic_activity
2717	Unigene42071_All	similar to ubiquitin conjugating enzyme E2 [*Cyanidioschyzon merolae s*train 10D]	gi|449,018,406|dbj|BAM81808.1|	binding catalytic_activity
4135	CL4653_Contig2_All	peptidase C12, ubiquitin carboxyl-terminal hydrolase 1 [*Coccomyxa subellipsoidea C-169*]	gi|384,249,810|gb|EIE23291.1|	cell cell_part
3660	CL649_Contig6_All	Rubisco activase [*Coccomyxa subellipsoidea C-169*]	gi|384,253,108|gb|EIE26583.1|	binding
1212	CL492_Contig1_All	bifunctional sensory photoreceptor [*Volvox carteri* f*_ nagariensis*]	gi|302,844,634|ref.|XP_002953857.1|	Energy convert
622	gi|315,319,013|gb|ADU04518_1|	LhcSR [*U. prolifera*]	gi|315,319,013|gb|ADU04518.1|	Energy convert
621	gi|158,277,339|gb|EDP03108_1|	serine/threonine protein kinase 2 [*Chlamydomonas reinhardtii*]	gi|159,472,056|ref.|XP_001694172.1|	Signal
2807	Unigene25558_All	calcium-dependent protein kinase [*Chlamydomonas reinhardtii*]	gi|159,464,829|ref.|XP_001690644.1|	Signal
329	CL2614_Contig3_All	ArfB-family small GTPase [*Coccomyxa subellipsoidea* C-169]	gi|384,244,739|gb|EIE18237.1|	Signal
1431	Unigene44820_All	ras-related gtp-binding protein [*Micromonas sp*_ RCC299]	gi|255,075,683|ref.|XP_002501516.1|	Signal
2089	Unigene4025_All	minus strand ras-related protein Rab2BV [*Coccomyxa subellipsoidea* C-169]	gi|307,102,647|gb|EFN50917.1|	Signal
3066	gi|158,271,373|gb|EDO97193_1|	ARF-like GTPase [*Chlamydomonas reinhardtii]*	gi|159,488,198|ref.|XP_001702104.1|	Signal
63	Unigene7624_All	NDPK2a nucleotide diphosphate kinase 2 protein [*Physcomitrella patens* subsp*_ patens*]	gi|168,020,398|ref.|XP_001762730.1|	Signal
3025	CL5370_Contig1_All	adenylate kinase [*Coccomyxa subellipsoidea* C-169]	gi|384,247,647|gb|EIE21133.1|	Signal
1512	CL3463_Contig1_All	calcium-dependent protein kinase(CDPK)[*Coccomyxa subellipsoidea* C-169]	gi|384,245,250|gb|EIE18745.1|	Signal
3467	CL3910_Contig4_All	calcium-dependent protein kinase(CDPK)[*Volvox carteri* f*_ nagariensis*]	gi|384,245,250|gb|EIE18745.1|	Signal
4337	Unigene26962_All	calcium-dependent protein kinase(CDPK) [*Chlamydomonas reinhardtii*]	gi|159,464,829|ref.|XP_001690644.1|	Signal
3383	CL7329_Contig1_All	histidine-aspartic acid phosphotransferase [*Chlamydomonas reinhardtii*]	gi|159,463,834|ref.|XP_001690147.1|	Signal
1545	CL4735_Contig1_All	kinase-like protein-SnRK2 [*Coccomyxa subellipsoidea* C-169]	gi|384,245,989|gb|EIE19481.1|	Signal
4259	CL6485_Contig1_All	metacaspase type II [*Chlamydomonas reinhardtii*]	gi|159,467,293|ref.|XP_001691826.1|	catalytic_activity
1524	gi|28,076,780|gb|AAO31578_1|	ribulose-1,5-bisphosphate carboxylase/oxygenase large subunit, partial (chloroplast) [*Ulva lactuca*]	gi|28,076,780|gb|AAO31578.1|	Carbohydrate transport and metabolism
1650	gi|145,567,455|gb|ABP82085_1|	ribulose-1,5-bisphosphate carboxylase/oxygenase large subunit, partial (chloroplast) [*Ulva sp*_ WELT A027500]	gi|219,932,444|emb|CAR65339.1|	Carbohydrate transport and metabolism
3021	gi|158,282,407|gb|EDP08159_1|	triacylglycerol lipase-like protein(EDS1) [*Chlamydomonas reinhardtii*]	gi|159,476,166|ref.|XP_001696182.1|	Plant-pathogen interaction
Down-regulated				
397	CL206_Contig2_All	elongation factor 2-like [*Solanum lycopersicum*]	gi|356,525,774|ref.|XP_003531498.1|	Response to stimulus
1080	Unigene25013_All	methionine sulfoxide reductase B [*Ulva fasciata*]	gi|197,631,364|gb|ACH70611.1|	Response to stimulus
1140	CL6594_Contig3_All	oxygen-evolving enhancer protein 1 precursor [*Volvox carteri* f_ *nagariensis*]	gi|302,846,662|ref.|XP_002954867.1|	Response to stimulus
1295	CL4649_Contig1_All	ferritin [*Ulva fasciata*]	gi|149,275,663|gb|ABR23157.1|	Response to stimulus
1603	Unigene17811_All	chloroplast stem-loop-binding protein [*Chlamydomonas reinhardtii]*	gi|159,464,355|ref.|XP_001690407.1|	Response to stimulus
1611	CL1851_Contig5_All	TPA_inf: chloroplast light-harvesting complex II protein precursor Lhcbm5 [*Acetabularia acetabulum*]	gi|145,079,356|tpg|DAA05911.1|	Response to stimulus
1686	gi|158,277,735|gb|EDP03502_1|	Adenylosuccinate synthetase, chloroplastic;	gi|159,469,564|ref.|XP_001692933.1|	Response to stimulus
1762	CL891_Contig1_All	actin [*Ulva linza*]	gi|363,992,274|gb|AEW46682.1|	Response to stimulus
1828	Unigene18752_All	putative early light-inducible protein 2 [*Ulva linza*]	gi|380,468,161|gb|AFD61608.1|	Response to stimulus
1855	CL7525_Contig1_All	chloroplast glycerolipid omega-3-fatty acid desaturase [*Chlamydomonas reinhardtii*]	gi|159,462,866|ref.|XP_001689663.1|	Response to stimulus
2532	gi|1,181,548|gb|AAA86855_1|	Glyceraldehyde-3-phosphate dehydrogenase A, chloroplastic;	gi|159,463,282|ref.|XP_001689871.1|	Response to stimulus
2584	Unigene27523_All	minus strand plastocyanin precursor [*Ulva pertusa*]	gi|48,526,878|gb|AAT45616.1|	Response to stimulus
2923	gi|344,012|dbj|BAA02024_1|	photosystem II D1 protein (chloroplast) [*Pinus thunbergii*]	gi|7,524,753|ref.|NP_042347.1|	Response to stimulus
3498	Unigene37824_All	minus strand tubulin beta [*Nannochloropsis gaditana* CCMP526]	gi|422,295,792|gb|EKU23091.1|	Response to stimulus
3524	gi|158,271,667|gb|EDO97482_1|	porphobilinogen deaminase [*Chlamydomonas reinhardtii*]	gi|159,486,921|ref.|XP_001701485.1|	Response to stimulus
3644	CL438_Contig1_All	fructose-1,6-bisphosphatase [*Chlamydomonas reinhardtii*]	gi|159,465,323|ref.|XP_001690872.1|	Response to stimulus
3749	CL930_Contig2_All	glutamate-1-semialdehyde aminotransferase [*Chlamydomonas reinhardtii*]	gi|384,247,824|gb|EIE21309.1|	Response to stimulus
3798	gi|288,816,175|gb|ADC54911_1|	oxygen-evolving enhancer protein, partial [*Ulva* sp_ EE2]	gi|288,816,175|gb|ADC54911.1|	Response to stimulus
1056	gi|323,320,892|gb|ADX36432_1|	ribulose-1,5-bisphosphate carboxylase/oxygenase large subunit, partial (chloroplast) [*U. prolifera*]	gi|32,492,888|gb|AAP85534.1|	Carbohydrate transport and metabolism
1767	gi|145,567,588|gb|ABP82151_1|	ribulose-1,5-bisphosphate carboxylase/oxygenase large subunit, partial (chloroplast) [*Ulva* sp*_* WELT A027397]	gi|159,475,703|ref.|XP_001695958.1|	Carbohydrate transport and metabolism
1854	Unigene19826_All	minus strand pyruvate orthophosphate dikinase *[Ulva linza*]	gi|255,076,895|ref.|XP_002502111.1|	Carbohydrate transport and metabolism
2044	CL7324_Contig1_All	glyceraldehyde-3-phosphate dehydrogenase, cytosolic-like [*Cucumis sativus*]	gi|384,245,592|gb|EIE19085.1|	Carbohydrate transport and metabolism
2532	gi|1,181,548|gb|AAA86855_1|	glyceraldehyde-3-phosphate dehydrogenase A, chloroplastic;	gi|323,320,892|gb|ADX36432.1|	Carbohydrate transport and metabolism
2561	CL2687_Contig1_All	minus strand pyruvate orthophosphate dikinase [*U. prolifera*]	gi|255,076,279|ref.|XP_002501814.1|	Carbohydrate transport and metabolism
2674	gi|145,567,931|gb|ABP82322_1|	ribulose-1,5-bisphosphate carboxylase/oxygenase large subunit, partial (chloroplast) [*Ulva sp*_ WELT A027743]	gi|449,475,837|ref.|XP_004154566.1|	Carbohydrate transport and metabolism
2812	CL1803_Contig1_All	phosphoglycerate kinase [*Chlamydomonas reinhardtii]*	gi|159,463,282|ref.|XP_001689871.1|	Carbohydrate transport and metabolism
2938	gi|342,316,037|gb|AEL22116_1|	pyruvate orthophosphate dikinase [*Ulva linza*]	gi|378,940,382|gb|AFC75637.1|	Carbohydrate transport and metabolism
3212	gi|145,568,009|gb|ABP82361_1|	ribulose-1,5-bisphosphate carboxylase/oxygenase large subunit, partial (chloroplast) [*Ulva* sp_ WELT A027348]	gi|145,568,333|gb|ABP82523.1|	Carbohydrate transport and metabolism
3644	CL438_Contig1_All	fructose-1,6-bisphosphatase [*Chlamydomonas reinhardtii*]	gi|342,316,037|gb|AEL22116.1|	Carbohydrate transport and metabolism
3884	CL5964_Contig1_All	minus strand starch branching enzyme II [*Parachlorella kessleri*]	gi|194,396,261|gb|ACF60500.1|	Carbohydrate transport and metabolism
1080	Unigene25013_All	methionine sulfoxide reductase B [*Ulva fasciata*]	gi|197,631,364|gb|ACH70611.1|	Posttranslational modification, protein turnover, chaperones
2047	gi|300,259,068|gb|EFJ43299_1|	molecular chaperone [*Volvox carteri* f_ *nagariensis*]	gi|302,848,253|ref.|XP_002955659.1|	Posttranslational modification, protein turnover, chaperones
2273	gi|158,279,996|gb|EDP05755_1|	signal peptide peptidase, partial [*Chlamydomonas reinhardtii*]	gi|159,464,533|ref.|XP_001690496.1|	Posttranslational modification, protein turnover, chaperones
2744	Unigene5984_All	thioredoxin x (ISS) [*Ostreococcus tauri*]	gi|308,804,023|ref.|XP_003079324.1|	Posttranslational modification, protein turnover, chaperones
3388	Unigene9950_All	thioredoxin dependent peroxidase [*Chlamydomonas reinhardtii*]	gi|159,489,214|ref.|XP_001702592.1|	Posttranslational modification, protein turnover, chaperones
4415	CL3087_Contig4_All	molecular chaperone [*Volvox carteri* f_ *nagariensis*]	gi|302,839,709|ref.|XP_002951411.1|	Posttranslational modification, protein turnover, chaperones
141	CL451_Contig4_All	diadenosine tetraphosphatase and related serine/threonine protein phosphatases[*Chlorella variabilis*]	gi|307,103,797|gb|EFN52054.1|	Signal transduction mechanisms
242	CL5191_Contig2_All	serine/threonine protein phosphatase [*Guillardia theta* CCMP2712]	gi|348,671,120|gb|EGZ10941.1|	Signal transduction mechanisms
250	Unigene7524_All	protein serine/threonine phosphatase 2C [*Coccomyxa subellipsoidea* C-169]	gi|307,104,872|gb|EFN53124.1|	Signal transduction mechanisms
3622	CL6423_Contig1_All	minus strand protein tyrosine phosphatase [*Ectocarpus siliculosus*]	gi|299,116,693|emb|CBN74838.1|	Signal transduction mechanisms
4212	CL4299_Contig1_All	serine/threonine protein phosphatase[*Volvox carteri* f*_ nagariensis*]	gi|302,851,958|ref.|XP_002957501.1|	Signal transduction mechanisms
2947	Unigene205_All	phytoene dehydrogenase and related proteins[*Chlorella variabilis*]	gi|384,249,750|gb|EIE23231.1|	Secondary metabolites biosynthesis, transport
3019	gi|158,273,526|gb|EDO99315_1|	cytochrome P450, CYP85 clan, partial [*Chlamydomonas reinhardtii*]	gi|159,481,496|ref.|XP_001698815.1|	Secondary metabolites biosynthesis
3998	CL7806_Contig1_All	carotenoid cleavage dioxygenase [*Chlamydomonas reinhardtii*]	gi|159,474,908|ref.|XP_001695565.1|	Secondary metabolites biosynthesis
4156	CL6885_Contig2_All	phytoene dehydrogenase and related proteins [*Volvox carteri* f_ *nagariensis*]	gi|302,835,624|ref.|XP_002949373.1|	Secondary metabolites biosynthesis
397	CL206_Contig2_All	elongation factor 2-like [*Solanum lycopersicum*]	gi|356,525,774|ref.|XP_003531498.1|	Translation
4324	gi|452,119,419|gb|AGG09538_1|	elongation factor Tu, partial (chloroplast) [*U. prolifera*]	gi|452,119,405|gb|AGG09531.1|	Translation
756	Unigene5495_All	oxidoreductase [*Coccomyxa subellipsoidea* C-169]	gi|384,249,901|gb|EIE23381.1|	Energy production and conversion
1323	CL514_Contig1_All	glutathione reductase [*Ulva fasciata*]	gi|82,658,844|gb|ABB88584.1|	Energy production and conversion
1554	CL3939_Contig2_All	glutathione reductase [*Ulva fasciata*]	gi|82,658,844|gb|ABB88584.1|	Energy production and conversion
1808	Unigene43735_All	photosystem I subunit VII (chloroplast) [*Trebouxiophyceae* sp_ MX-AZ01]	gi|357,467,753|ref.|XP_003604161.1|	Energy production and conversion
2584	Unigene27523_All	plastocyanin precursor [*Ulva pertusa*]	gi|48,526,878|gb|AAT45616.1|	Energy production and conversion

### GO function analysis of DEPs

**Fig. 2:**
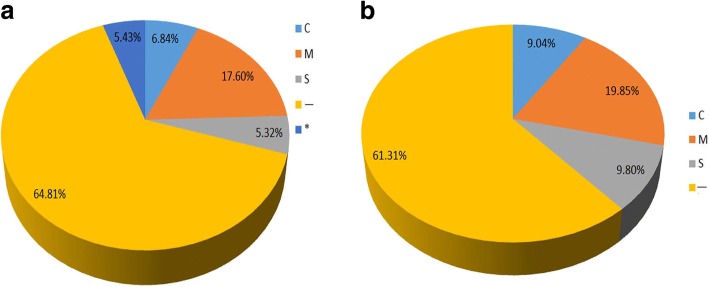
Subcellular localization of the differentiallyexpressed proteins under heat stress. **a** Subcellular localization of the up-regulated proteins **b** Subcellular localization of the down-regulated proteins Note: C: Chloroplast, i.e. the sequence contains cTP, a chloroplasttransit peptide; M: Mitochondrion, i.e. the sequence contains mTP, amitochondrial targeting peptide; S: Secretory pathway, i.e. the sequencecontains SP, a signal peptide; −: other location

GO analysis was conducted on DEPs using the Blast2GO program; 469, 481 and 473 DEPs were successfully assigned to the biological processes, molecular function, and cellular components GO categories, respectively. The distribution of GO terms (the second level) for the three categories is shown in Table 2. For the biological process category, the most abundant groups were metabolic process (42.20%), cellular process (39.40%), and translation (24.90%). In addition, 126 DEPs belonged to response to stimulus (26.86%), of which 86 DEPs were up-regulated and 40 DEPs were down-regulated. For the cellular component category, cells (63.59%), cell parts (63.59%), and organelles (51.79%) were the most abundant groups. In the molecular function category, most of the DEPs were classified into catalytic activity (58.07%) and binding functions (45.70%); antioxidant activity only accounted for 0.72% of the DEPs (Table 2). Furthermore, subcellular localization of the 790 up-regulated characterized proteins showed that 54 proteins (6.84%) were located in the chloroplast, 139 proteins (17.60%) were assigned to the mitochondria, 42 proteins (5.32%) belonged to the secretory pathway, and 512 proteins (12%) were classified as belonging to other locations. Forty-three of the DEPs had unknown locations (Fig. 2). Furthermore, subcellular localization analysis of the 433 down-regulated proteins showed that quite many chloroplast proteins and mitochondria proteins are related to the thermotolerance of *U. prolifera*.

**Table 2:** GO analysis of DEPs (UpHT vs UpC) (top15)

Gene Ontology term	Cluster frequency	Protein frequency of use	*P*-value
translation	117 out of 469 genes, 24.9%	192 out of 1588 genes, 12.1%	3.420412e-22
protein metabolic process	198 out of 469 genes, 42.2%	424 out of 1588 genes, 26.7%	7.955088e-19
cellular protein metabolic process	185 out of 469 genes, 39.4%	389 out of 1588 genes, 24.5%	2.034726e-18
gene expression	131 out of 469 genes, 27.9%	251 out of 1588 genes, 15.8%	1.381491e-16
cellular macromolecule biosynthetic process	136 out of 469 genes, 29.0%	275 out of 1588 genes, 17.3%	1.195581e-14
response to stimulus	126 out of 469 genes, 26.86%	465 of 1588 in all the Protein	1.921323e-6
structural constituent of ribosome	93 out of 481 genes, 19.3%	114 out of 1743 genes, 6.5%	1.412589e-35
structural molecule activity	104 out of 481 genes, 21.6%	142 out of 1743 genes, 8.1%	2.312654e-32
rRNA binding	31 out of 481 genes, 6.4%	33 out of 1743 genes, 1.9%	6.624359e-16
RNA binding	73 out of 481 genes, 15.2%	131 out of 1743 genes, 7.5%	1.526571e-12
nucleic acid binding	98 out of 481 genes, 20.4%	224 out of 1743 genes, 12.9%	1.810790e-08
ribonucleoprotein complex	138 out of 473 genes, 29.2%	187 out of 1477 genes, 12.7%	1.942048e-36
ribosome	122 out of 473 genes, 25.8%	166 out of 1477 genes, 11.2%	1.634089e-31
non-membrane-bounded organelle	165 out of 473 genes, 34.9%	281 out of 1477 genes, 19.0%	4.775761e-25
intracellular non-membrane-bounded organelle	165 out of 473 genes, 34.9%	281 out of 1477 genes, 19.0%	4.775761e-25
macromolecular complex	214 out of 473 genes, 45.2%	409 out of 1477 genes, 27.7%	3.124677e-24

### COG function class of DEPs

**Fig. 3:**
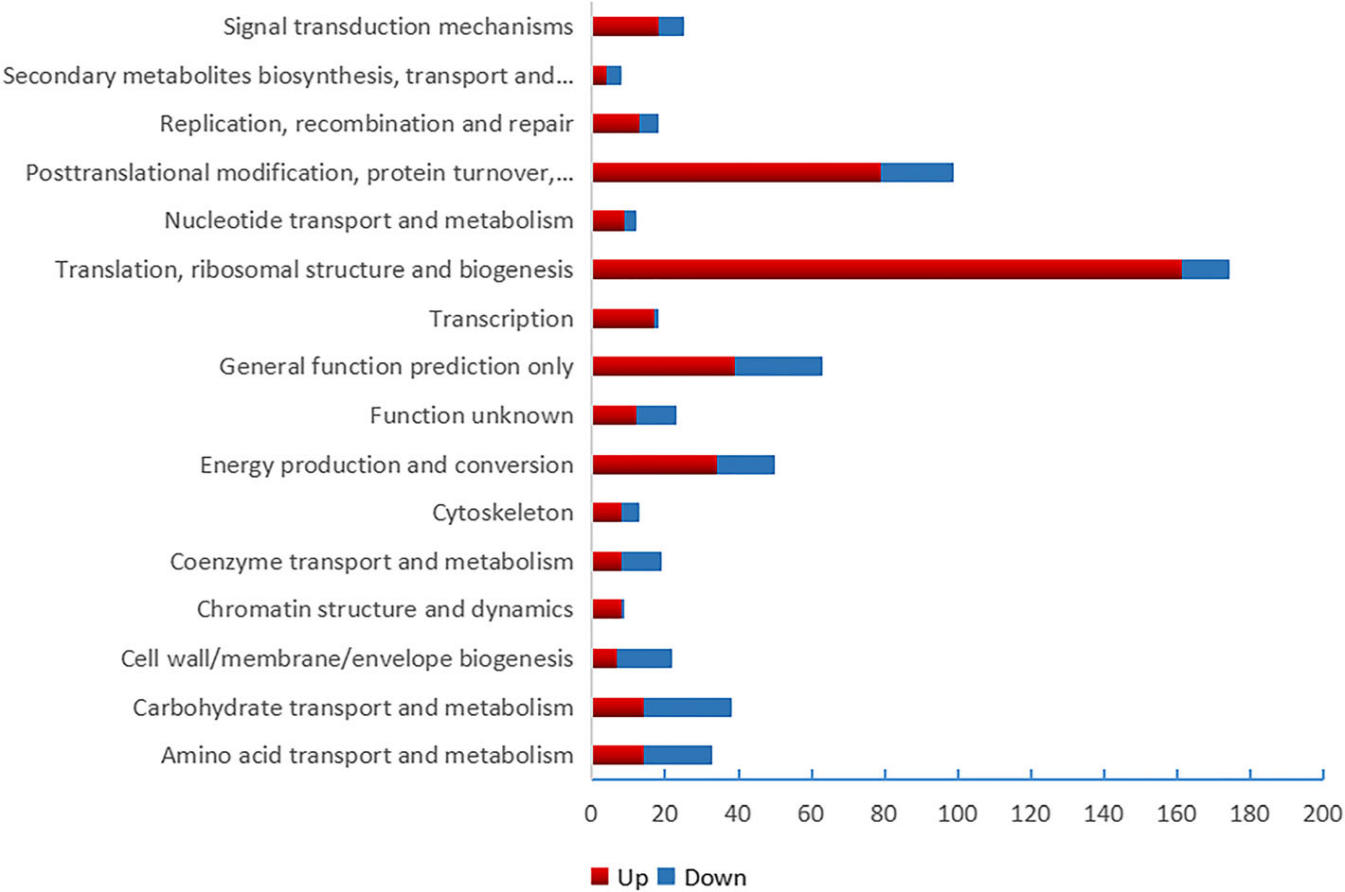
COG function analysis DEPs of UpHT vs UpC. Colour in red indicated up-regulated proteins number, blue indicated down-regulated proteins number

In the COG function analysis, the largest category was “translation, ribosomal structure and biogenesis” (161 DEPs up-regulated, 13 DEPs down-regulated), followed by “post-translational modification, protein turnover, molecular chaperone” (79DEPs up-regulated and 20 DEPs down-regulated) and “general function prediction only” (39 DEPs up-regulated and 24 DEPs down-regulated) (Fig. 3).

### KEGG pathway analysis of DEPs

**Fig. 4:**
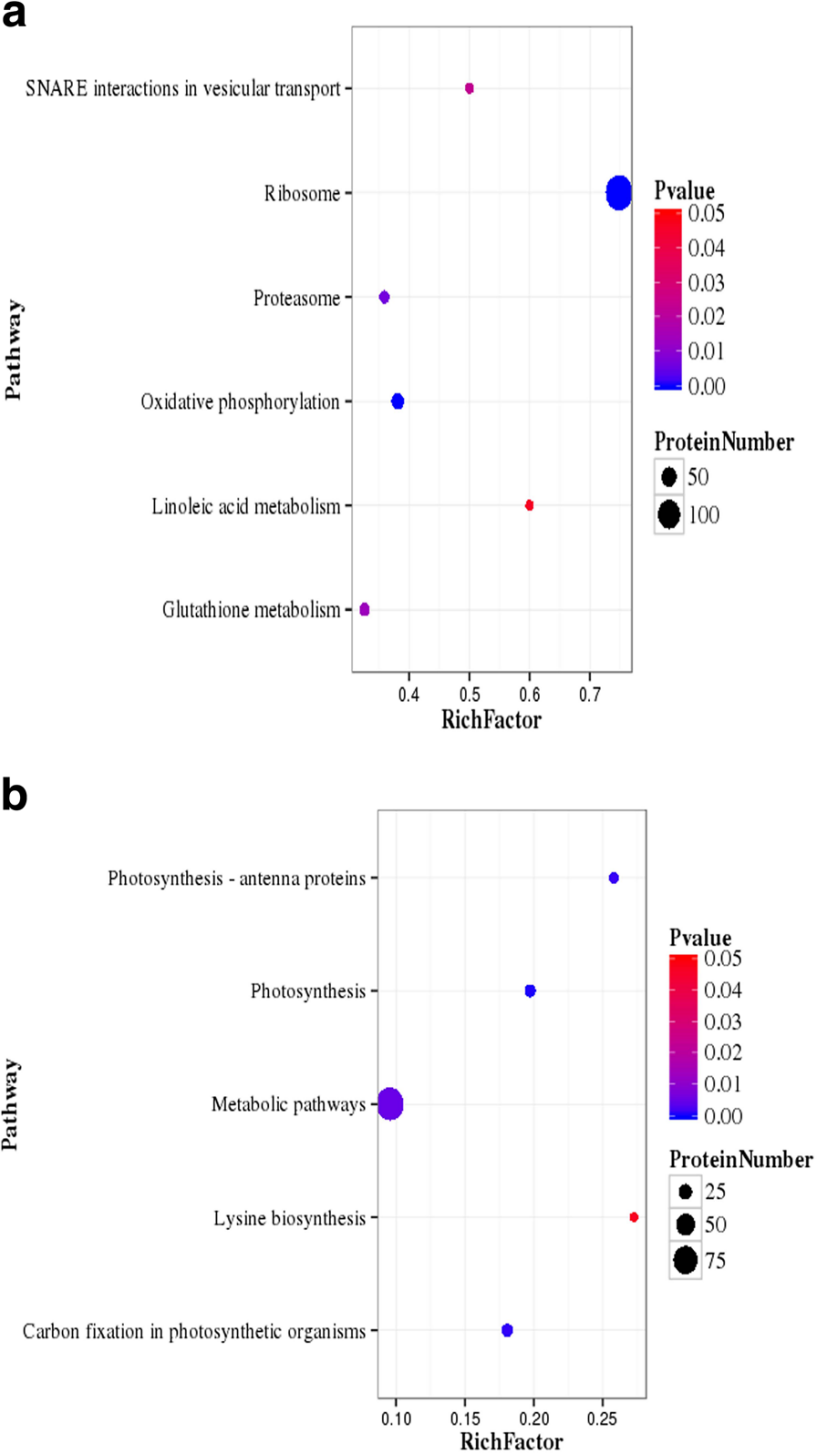
Pathway enrichment statistical scatter plot of DEPs. **a** UpHT vs UpC up-regulated proteins **b** UpHT vs UpC down-regulated proteins. The vertical axis represents the name of the pathway; the horizontal axis represents the pathway corresponding Rich factor. The colour indicated *p* value. Rich factor refers to the ratio of the number of differentially expressed genes in the pathway and the number of all annotated genes in the pathway. Higher Rich factors indicate greater degrees of enrichment. Q values are often completed after multiple hypothesis testing with corrected *P* value values ranging from 0 to 0.05. The closer they are to zero, the more significant the enrichment

KEGG pathway analysis of DEPs were conducted online at http://www.genome.jp/kegg/kegg2.html; 557 up-regulated proteins were mapped to 97 KEGG pathways, and the top 15 pathways are listed in Table 3. The pathway enrichment analysis showed that the most significantly enriched pathways among the 557 up-regulated proteins were ribosome (134, 24.05%), oxidative phosphorylation (32, 5.75%), proteasome (14, 2.51%), glutathione metabolism (15, 2.69%), SNARE interactions in vesicular transport (5, 0.89%) and linoleic acid metabolism (3, 0.53%) (Fig. 4a). Two hundred and thirty-three down-regulated proteins were enriched in photosynthesis (15, 6.44%), carbon fixation in photosynthesis organisms (15, 6.44%), photosynthesis antenna proteins (8, 8.43%), metabolic pathways (91, 39.06%) and lysine biosynthesis (3, 1.2%) (Fig. 4b). At present, glutathione S-transferase, heat shock proteins, MnSOD and ubiquitin-related proteins were up-regulated. The results indicated that upon high-temperature stimulus, *U. prolifera* induces defence mechanisms, and the expression of heat shock proteins and antioxidant-associated proteins is increased.

**Table 3:** KEGG pathway of DEPs(top15)(UpHT vs UpC)

Pathway	DEPs with pathway annotation	All Proteins with pathway annotation (3046)	P value	Pathway ID
Up-regulated				
Ribosome	134	179	2.73E-66	ko03010
Oxidative phosphorylation	32	84	1.18E-05	ko00190
Proteasome	14	39	0.006672349	ko03050
Glutathione metabolism	15	46	0.01352303	ko00480
SNARE interactions in vesicular transport	5	10	0.0225611	ko04130
Linoleic acid metabolism	3	5	0.04546205	ko00591
Protein processing in endoplasmic reticulum	23	90	0.05135553	ko04141
Phagosome	13	45	0.05413349	ko04145
Spliceosome	25	101	0.06097134	ko03040
Non-homologous end-joining	2	3	0.08799354	ko03450
RNA transport	22	95	0.1338129	ko03013
Porphyrin and chlorophyll metabolism	12	47	0.1356993	ko00860
Taurine and hypotaurine metabolism	1	1	0.1828628	ko00430
Vancomycin resistance	1	1	0.1828628	ko01502
Plant-pathogen interaction	11	48	0.2512615	ko04626
Down-regulated				
Photosynthesis	15	76	0.00047354	ko00195
Carbon fixation in photosynthetic organisms	15	83	0.001247104	ko00710
Photosynthesis - antenna proteins	8	31	0.001746649	ko00196
Metabolic pathways	91	952	0.005208609	ko01100
Lysine biosynthesis	3	11	0.04605148	ko00300
Betalain biosynthesis	1	1	0.07649376	ko00965
Carotenoid biosynthesis	4	23	0.09360185	ko00906
Selenocompound metabolism	3	17	0.1357731	ko00450
Monobactam biosynthesis	2	9	0.146953	ko00261
Degradation of aromatic compounds	1	2	0.1471594	ko01220
Tyrosine metabolism	3	18	0.1542241	ko00350
Tryptophan metabolism	3	19	0.1734048	ko00380
One carbon pool by folate	3	19	0.1734048	ko00670
Insulin resistance	4	29	0.1770334	ko04931

### Expression analysis of DEPs in response to stimulus

Abiotic stresses such as high temperature, low temperature, and drought induced plant defence mechanisms, including the expression of antioxidant enzymes to regulate their adaptation [33]. In higher plants, the signal response to high-temperature stress involves a reduction in the synthesis of normal proteins and is accompanied by accelerated expression of heat-responsive genes and HSPs. Reactive oxygen species (ROS) refer to free radicals, including hydrogen peroxide (H_2_O_2_), singlet oxygen (O_2_^−^), and hydroxyl radicals (OH^−^). Excessive amounts of ROS can damage macromolecules and cell membranes. To remove toxic ROS, algae have developed a number of antioxidant systems that serve as protective mechanisms, namely, superoxide dismutases (SOD), catalases, peroxidases, thioredoxin, and glutathione [34]. In the present study, high-temperature stress can lead to protein misfolding and inactivation and desiccation of plant cells. Heat shock proteins are important molecular chaperones of the Hsp family, which prevents the aggregation of misfolded proteins. Significant up-regulation was observed for the heat shock protein family, which included 90 (CL5666_Contig1_All, 1.5-fold), 90C (Unigene40313_All, 1.78-fold), 70 (gi|219764948|emb|CAW63927_1|, 1.4-fold), ClpB chaperone, Hsp100 family (CL517_Contig2_All, 1.64-fold). Therefore, it could play a role in protecting the protein structure early in the stress response. DNA repair protein Rad50 (CL6208_Contig1_All, 1.22-fold) were identified and significantly up-regulated under high temperature. It means that the DNA repair protein Rad50 played an important role in DNA damage induced by high temperature. We also determined that antioxidant proteins exhibited highly dynamic changes in response to the high-temperature stimulus. Reactive oxygen or nitrogen species are generated in the plant cell during the extreme stress condition, which produces toxic compounds after reacting with the organic molecules. The glutathione-S-transferase (GST) enzymes play a significant role in detoxifying these toxins and help in their excretion or sequestration; the enzymes catalyse reduction of glutathione (GSH; a tripeptide Glu-Cys-Gly) by electrophilic and hydrophobic toxic molecules that are generated under stress to convert them to non-toxic and soluble conjugates [35]. The expression of glutathione S-transferase (Unigene15839_All,1.45-fold), MnSOD (CL6691_Contig2_All, 1.42-fold), ascorbate peroxidase (CL1454_Contig2_All,1.28-fold) and polyubiquitin (Unigene6725_All, 1.54-fold) were up-regulated. In addition, the expression of catalase-peroxidase (CL5101_Contig1_All, 0.66-fold), glutathione reductase (CL514_Contig1_All, 0.77-fold), and thioredoxin-dependent peroxidase (Unigene9950_All, 0.77-fold) were down-regulated.

In addition, the 26S proteasome regulatory subunit is a component of the hexameric ring of AAA-ATPases that forms the base of the 19S regulatory particle (RP). This subunit plays specific roles in plant proteasomes by helping to promote the assembly of the RP with the 20S core protease (CP) and gate the CP to prevent indiscriminate degradation of cytosolic and nuclear proteins. In plants, this subunit plays an important role in diverse processes that include shoot and root apical meristem maintenance, cell size regulation, and stress responses [36]. In the present study, the 26S proteasome regulatory subunit (CL1786_Contig3_All, CL2484_Contig2_All, 1.3 or 1.32-fold) was induced under high-temperature stress. The result is consistent with the observation that the expression of the 26S proteasome subunit RPN10 is upregulated by salt stress in *Dunaliella viridis* [37]. The result indicated that the 26S proteasome regulatory subunit played a key role in stress tolerance. Programmed cell death (PCD) is an important biological phenomenon. Algae can activate PCD when exposed to external pathogens and other stresses. Metacaspases often play an important role in plant PCD, and a large number of experiments have shown that metacaspases are involved in plant PCD. In PCD, type II metacaspase was found to be transported from the cytoplasm to the nucleus, which promoted chromosome degradation. In the present study, mov34-domain-containing protein (Unigene14048_All, 1.33-fold) and metacaspase type II (CL6485_Contig1_All, 1.25-fold) were up-regulated. The results indicated that the high-temperature stimulus accelerated the accumulation of ROS, inhibited the growth of *U. prolifera*, induced the expression of partial stress related proteins, and alleviated the stimulation. This process is consistent with the results that are reported for the brown alga *Ectocarpus siliculosus* [38].

### Expression analysis of DEPs that are involved in the oxidative phosphorylation pathway

**Fig. 5:**
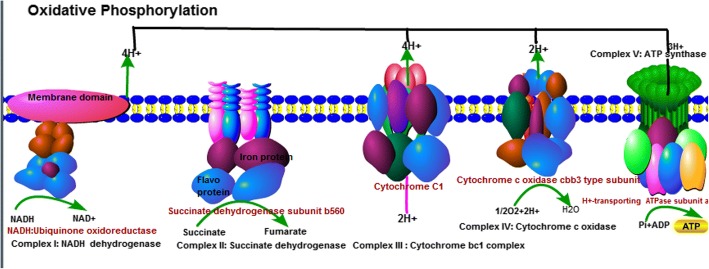
The oxidative phosphorylation pathway

**Fig. 6:**
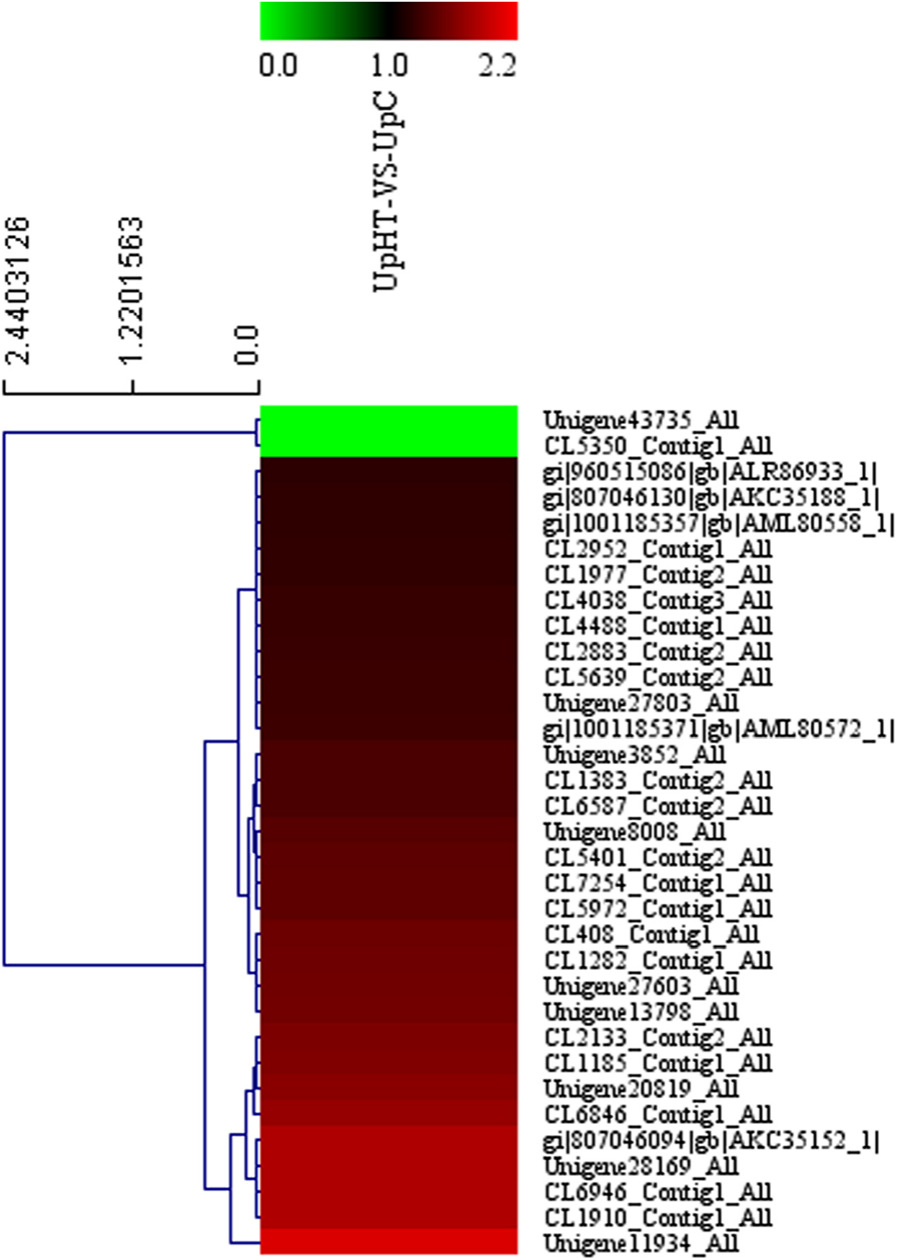
The cluster of oxidative phosphorylation pathway(UpHT vs UpC). Red indicated the expression of protein up-regulated, green indicated the expression of protein down-regulated

The oxidative phosphorylation (OXPHOS) process comprises an electron-transfer chain (ETC) that is driven by substrate oxidation and is coupled to the synthesis of ATP through an electrochemical transmembrane gradient. Oxidative phosphorylation is a vital part of metabolism, it also produces reactive oxygen species such as superoxide and hydrogen peroxide, which leads to propagation of free radicals, damages cells and contributes to disease, and possibly ageing. The previous study showed that low temperature, UVB radiation and phoxim-induced increase in oxidative phosphorylation gene [39–41]. In the present study, 34DEPs were mapped to the oxidative phosphorylation pathway (Figs. 5 and 6). The expression of 32 DEPs were found to be increased, and 2 DEPs were down-regulated. NADH:ubiquinone oxidoreductase (CL6587_Contig2_All), succinate dehydrogenase (CL2133_Contig2_All), cytochrome C oxidase subunit (CL1977_Contig2_All), and ATP synthesis subunit (gi|807046094|gb|AKC35152_1) were all significantly up-regulated by 1.36 fold, 1.59 fold, 1.23 fold, and 1.83 fold, respectively. The results are consistent with previous results. These results indicated that high-temperature induction enhanced the activity of the ETC complex in the mitochondria. The imbalance between the activity changes of ETC may lead to the accumulation of reactive oxygen species.

### Expression analysis of DEPs involved in phytohormone and signal transduction pathways

**Fig. 7:**
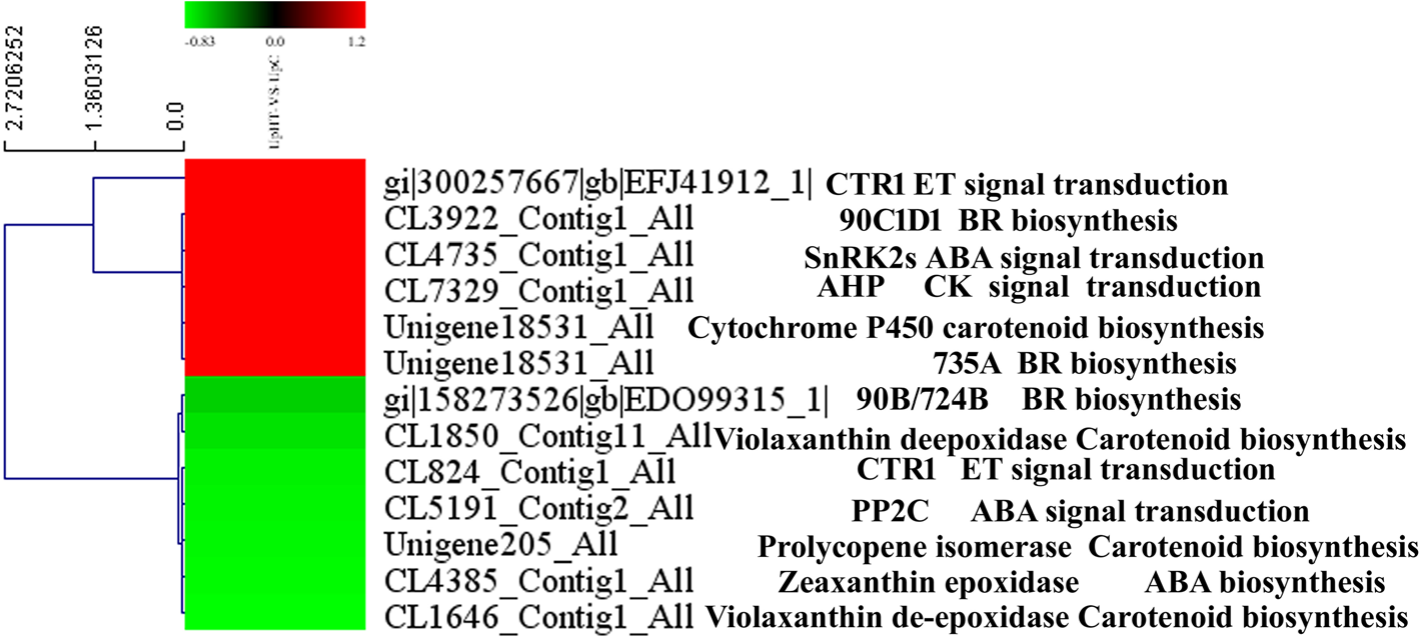
The cluster of plant hormone biosynthesis and signal transduction related proteins(UpHT vs UpC). Red indicated the expression of protein up-regulated, green indicated the expression of protein down-regulated

Studies have investigated the expression of the hormone-related proteins that were changed under abiotic stress conditions [42]. In the present study, expression pattern analysis showed that the high-temperature stimulus changed the expression of key enzymes involved in phytohormone signal transduction and hormone biosynthesis. Approximately 12 DEPs were identified in phytohormone signal transduction (Fig. 7). In carotenoid biosynthesis, cytochrome P450 (Unigene18531_All) was significantly up-regulated by 1.23 fold. The expression of violaxanthin de-epoxidase-related protein (CL1646_Contig1_Alland CL1850_Contig11_All), prolycopene isomerase (Unigene205_All) and zeaxanthin epoxidase (CL4385_Contig1_All) were down-regulated by 0.83 fold, 0.74 fold, 0.81 fold, and 0.82 fold compared to the control. In brassinosteroid (BR) biosynthesis, molecular and biochemical studies revealed that two different cytochrome P450s, CYP90B2 and CYP724B1/D11, are the rate-limiting step of brassinosteroid biosynthesis. Three significant DEPs that were mapped to the BR synthesis pathway the expression of cytochrome P450s 90B/724B (gi|158273526|gb|EDO99315_1|, 0.69-fold) were decreased. The expression of 90C1D1 (CL3922_Contig1_All, 1.29-fold) and CYP735A (Unigene18531_All, 1.23-fold) were up-regulated. CYP735A encodes cytokinin hydroxylases that catalyse iP-nucleotide converts to trans-Zeatin. The signalling transduction of the cytokinin pathway and abscisic acid (ABA) pathway played an important role in the growth and development of plants and the regulation of stress resistance. AHP (Histidine-containing phosphotransfer protein) mainly functions as two-component phosphorelay mediators between cytokinin sensor histidine kinases and response regulators (B-type ARRs) and plays an important role in promoting cytokinin signal transduction via the multistep His-to-Asp phosphorelay. Biochemical and molecular genetic studies in *Arabidopsis* have identified protein phosphatase 2C (PP2C) enzymes as negative regulators in plant signal transduction processes [43]. In the absence of ABA, PP2C mainly inhibited the active state of SnRK2s protein. The inhibition of the active state of SnRK2s protein result in a decrease in downstream transcriptional regulation. ABA relieved the inhibition effect of PP2C on SnRK2s, activated the SnRK2s protein, and initiated downstream signal transduction. In the present study, the expression of the AHP protein (CL7329_Contig1_All, 1.25-fold) and SnRK2s (CL4735_Contig1_All, 1.27-fold) protein was up-regulated. However, the expression of PP2C (CL5191_Contig2_All, 0.8-fold) was down-regulated, which indicated that the signal transduction pathway of cytokinin and ABA was induced at a high temperature. The result is consistent with previous rzesearch [42, 43]. Plant growth is co-ordinately regulated by environmental and hormonal signals. Brassinosteroid (BR), cytokinin and ABA played essential roles in growth regulation via light and temperature; however, the interactions between hormone signal transduction and environmental signals remain poorly understood at the molecular level. In summary, the results indicated that phytohormone signal regulation played a key role in the high-temperature stimulus and enhanced cytokinin and ABA signal transduction pathway. However, the regulation mechanism should be further considered.

### Expression analysis of DEPs involved in photosynthesis-related proteins

**Fig. 8:**
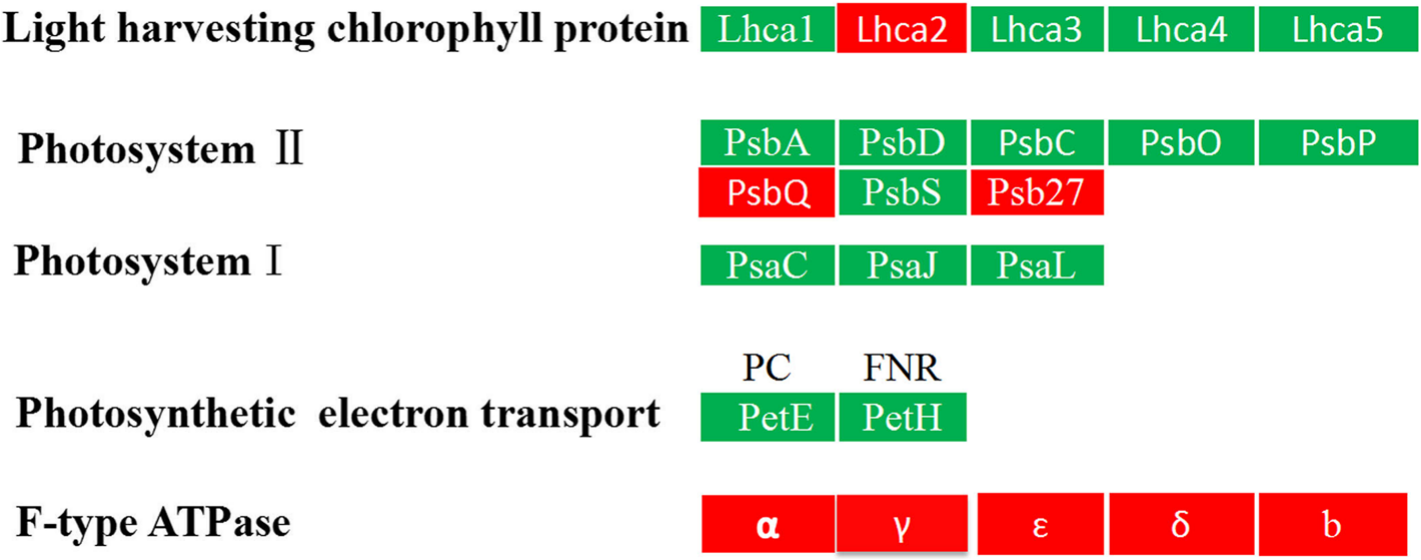
The expression of photosynthesis-related proteins (UpHT vs UpC). Red indicated the expression of protein up-regulated, green indicated the expression of protein down-regulated

Photosynthesis is very sensitive to high-temperature stress. Changes in environmental temperature are primarily reflected by photosynthesis, which triggers a response aimed at attaining the best possible performance under the new conditions. Several studies have shown that high-temperature stress can significantly inhibit photosynthesis [44, 45]. In green algae, light-harvesting chlorophyll protein complexes are the major light-harvesting complexes. These complexes include photosystem II (light-harvesting complex II and photosynthetic reaction centre) and photosystem I (LHCI and photosynthetic reaction centre). The light-harvesting complex is a complex of subunit proteins that may be part of a larger supercomplex of the photosystem, and the function is to collect more of the incoming light. In the previous study, LHCI of photosystem I *Chlamydomonas reinhardtii* is composed of nine genes. Three product complexes (Lhca1, 3 and 7) showed emission peaks in the range of 680–690 nm, thus accounting for the blue contribution observed in the native preparations. Three other complexes (Lhca5, 6 and 8) exhibit emission in the 695–700 nm range. This emission component is not clearly resolved in the fluorescence spectra of the native LHCI preparations, possibly due to energy transfer to the red forms of a neighbouring complex or due to a change in the absorption properties of these complexes, because of protein–protein interactions, when embedded in the PSI–LHCI supercomplex, as previously proposed [46]. Three more complexes, namely, Lhca2, 4 and 9, exhibited emission above 707 nm with the red-most form (715 nm) associated with Lhca2, which indicates that Lhca2 is responsible for the red-most emission in vivo. Up-regulation of Lhca4 and Lhca9 was observed under iron-deficiency conditions, and it correlates with a shift to the blue end of the emission spectrum (from 710 to 704 nm) and with a relative increase in intensity of the emission above 700 nm, compared to that for the emission below 700 nm [47]. This indicates an increase in the content of red forms that emit approximately 705 nm in the antenna, in agreement with the fluorescence characteristics of Lhca4 and Lhca9. Under the same conditions, down-regulation of Lhca5 was also detected, leadin to the suggestion that Lhca4 can substitute for Lhca5, which possibly provides a better sink for energy dissipation. The finding that these antennas contain the lowest energy forms supports the hypothesis. A similar effect was recently observed in higher plants, where Lhca5 could substitute for Lhca4 [48, 49]. In the present study, the LHCI subunits lhca1 (CL4203_Contig1_All, 0.72-fold) and lhca3 (CL654_Contig2_All, 0.8-fold) were down-regulated, and lhca2 (LhcSR, gi|315319013|gb|ADU04518_1|, 1.3-fold) was up-regulated. It may be possible that the decrease in the emission of blue light results in energy transfer to the red forms. Lhca2 is responsible for the red-most emission. The up-regulation of lhca2 indicated that the lowest energy forms were native organized. Lhca1 and lhca3 were unstable and underwent decomposition under high temperature. The results were consistent with that obtained by Naumann et al. [47]. In addition, the two most abundant light-harvesting complex II are Lhcb1 and Lhcb2, which make up light-harvesting complex (LHC) II trimers. They are also involved in facilitating state transitions, a process during which energy balance between photosystem (PS) II and I is achieved. In the present study, the LHCII subunits lhcb1 and lhcb2 were also down-regulated. Down-regulation of LHCII would significantly influence light absorbance and energy transfer in *U. prolifera* upon a high–temperature stimulus (Fig. 8). The results were consistent with the observation that salt stress induces a decrease in excitation energy transfer from phycobilisomes to photosystem II but an increase to photosystem I in the cyanobacterium *Spirulina platensis* [50]. Eighteen DEPs were mapped to the photosynthesis pathway, and the expression of 14 DEPs were down-regulated. Ferredoxin-NADP^+^ reductase (FNR) is one of the important enzymes involving in many biochemical and physiological metabolism processes, such as electron transfer, carbon dioxide fixation, nitrogen assimilation and antioxidation. Ferredoxin-NADP^+^reductase (Unigene9738_All, 0.82-fold and CL7087_Contig1_All, 0.81-fold) and photosystem I and photosystem II subunit proteins were identified and found to be decreased.

However, the expression of the chloroplast ATP synthase subunit that was encoded by 5 unigenes (gi|807,046,094|gb|AKC35152_1|, 1.82-fold) was up-regulated.

This result indicated that the high temperature inhibited photosynthesis of *U. prolifera* and induced stress-related LhcSR protein expression, which was consistent with the expression of LhcSR in *U. linza* in a study that was conducted by Dong et al. [51]. The electrons from PSI and PSII might transfer to oxygen, and thereby lead to substantial generation of reactive oxygen species. Enrichment of the oxidative phosphorylation pathway under cold treatment suggested that high–temperature induced ROS production might occur. However, this hypothesis still lacks experimental evidence.

### Expression analysis of DEPs involved in the carbon-fixation pathway

**Fig. 9:**
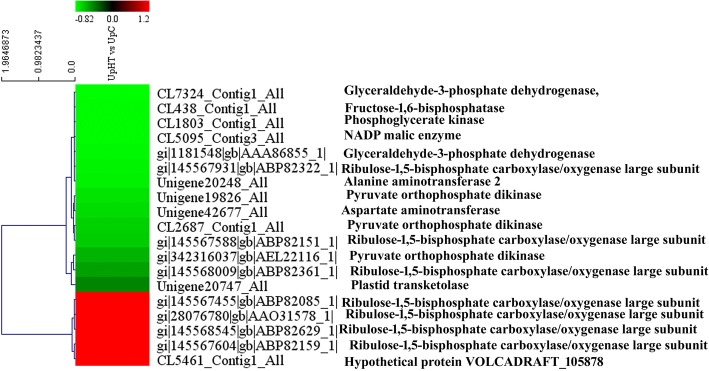
The cluster of carbon fixation related proteins (UpHT vs UpC). Red indicated the expression of protein up-regulated, green indicated the expression of protein down-regulated

According to CO_2_ assimilation mechanisms, plants can be classified into three types: C_3_, C_4_, and CAM. C_4_ plants have acquired a series of high-performance photosynthetic genes during evolution, which confer more efficient photosynthesis than that in C_3_ plants under adverse conditions such as high light intensity, temperature, and low CO_2_ concentration. In the present study, 19 DEPs were mapped to carbon fixation in photosynthetic organisms (Fig. 9). The expression of 14 DEPs was down-regulated, and that of 5 DEPs was up-regulated. In C_4_-dicarboxylic acid cycle, the key regulated enzymes aspartate aminotransferase (Unigene42677_All, 0.71-fold), pyruvate orthophosphate dikinase (Unigene19826_All, 0.73-fold), and NADP malic enzyme (CL5095_Contig3_All, 0.81-fold) were down-regulated. At the same time, pyruvate orthophosphate dikinase (Unigene19826_All, 0.73-fold) and NADP malic enzyme were also found to be down-regulated in CAM. Moreover, the expressions of plastid transketolase (Unigene20747_All, 0.43-fold), fructose-1,6-bisphosphatase (CL438_Contig1_All, 0.81-fold), glyceraldehyde-3-phosphate dehydrogenase (CL7324_Contig1_All, 0.72-fold) and phosphoglycerate kinase (CL1803_Contig1_All, 0.81-fold) were down-regulated. Ribulose-1,5-bisphosphatecarboxylase/oxygenase large subunit was found to be encoded by 7 DEPs, of which, 4 DEPs were up-regulated, and 3 DEPs were down-regulated. The result demonstrated that the expression of the key enzyme in the carbon-fixation pathway was down-regulated.

The activation of rubisco in vivo requires the presence of the regulatory protein rubisco activase. This enzyme facilitates the release of sugar phosphate inhibitors from rubisco catalytic sites thereby influencing carbamylation. Rubisco activase was used to explore the role of the enzyme in C_4_ photosynthesis at a high temperature. It is concluded that during short-term treatment at 40 °C, rubisco activase content is not the only factor that modulates rubisco carbamylation during C_4_ photosynthesis [52]. In the present study, rubisco activase (CL649_Contig6_All, 1.32-fold) was up-regulated; however, it is not the only factor. At the same time, soluble carbonic anhydrase precursor encoded by CL7120_Contig2_All was down-regulated, and CL6328_Contig1_All was up-regulated. In other words, to protect the energy balance, *U. prolifera* decreased photosynthesis and carbon fixation of metabolic activity under high-temperature conditions. However, increase in the expression of carbonic anhydrase precursor and rubisco activase play an important role in the acceleration to photosynthesis balance adjustment.

### Expression analysis of DEPs involved in the glutathione metabolism pathway

**Fig. 10:**
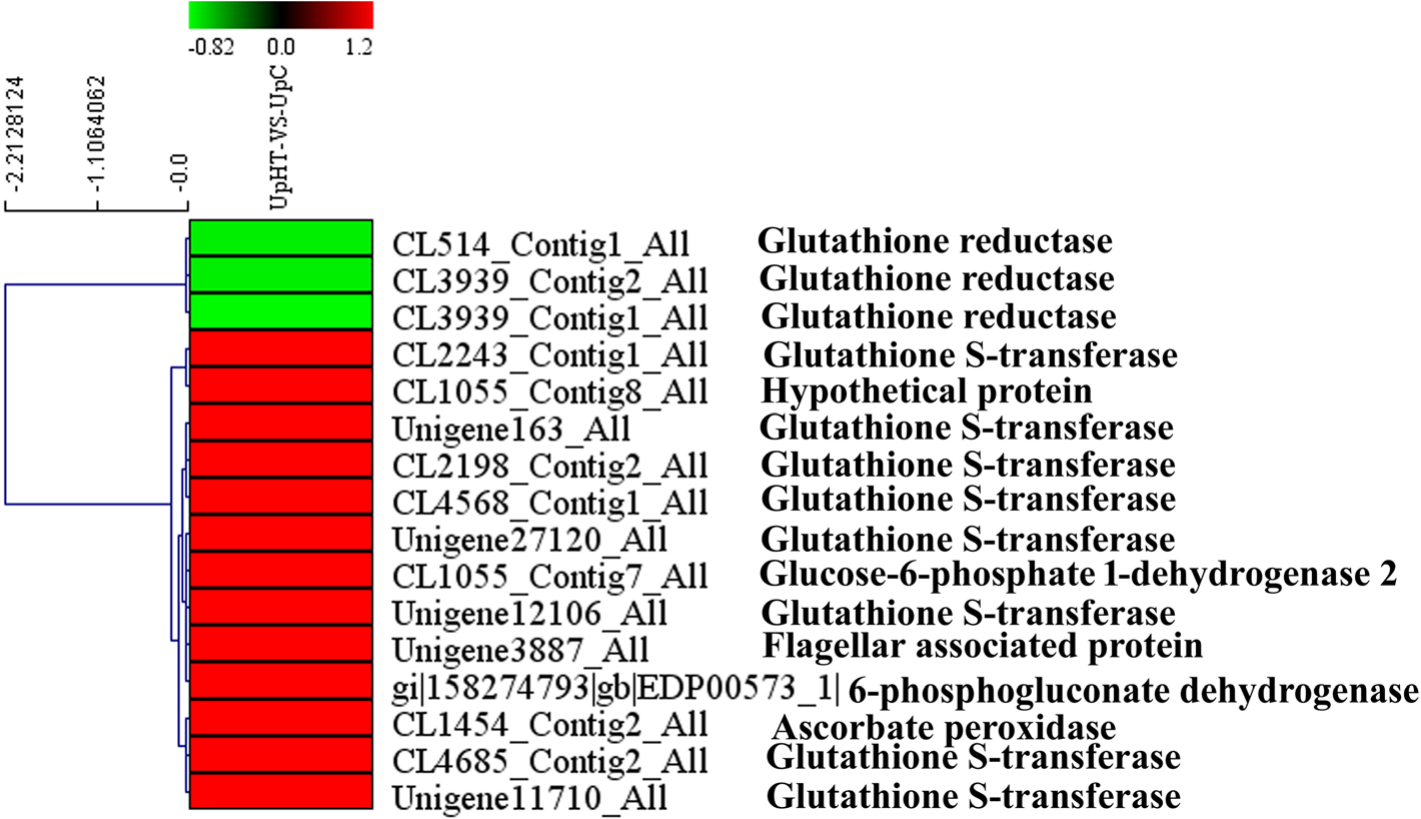
The cluster of glutathione metabolism related proteins (UpHT vs UpC). Red indicated the expression of protein up-regulated, green indicated the expression of protein down-regulated

A total of 18 DEPs were mapped to the glutathione metabolism pathway. The expression of key enzymes in glutathione metabolism pathway were observed to significantly change. Glutathione S-transferase (GST) catalyses the nucleophilic fusion of reduced glutathione (GSH; a tripeptide Glu-Cys-Gly) with electrophilic and hydrophobic toxic molecules, which were generated under stress, to convert them to non-toxic and soluble conjugates. GSTs play an important role in various metabolic pathways, and they are involved in detoxification of oxidative lipid peroxide metabolites, hormone metabolism, stress resistance, protection of cell oxidative stress injury, cell signal transduction and disease resistance [53–55]. Ascorbate peroxidase (APXs) catalyses the H_2_O_2_-dependent oxidation of ascorbate in plants, algae and certain cyanobacteria. Glutathione reductase (GR) catalyses the reduction of glutathione disulphide (GSSG) to the sulphydryl form glutathione (GSH), which is a critical molecule in resisting oxidative stress and maintaining the reducing environment inside the cell [56–58]. In the present study, APX (CL1454_Contig2_All, 1.28-fold), glutathione S-transferase (CL2243_Contig1_All, 1.53-fold), glucose-6-phosphate 1-dehydrogenase 2(CL2243_Contig1_All, 1.53-fold) and 6-phosphogluconate dehydrogenase (gi|158274793|gb|EDP00573_1|, 1.32-fold), decarboxylating(gi|158274793|gb|EDP00573_1|, 1.32-fold) were found to significantly increase. However, the expression of GR(CL514_Contig1_All, 0.77-fold) was down-regulated (Fig. 10). It is indicated that high temperature stimulus accumulated more ROS, induced the expression of resistance-related protein glutathione S-transferase and APX.

### Expression analysis of Ca^2+^-binding protein

Ca^2+^-dependent signalling processes are beneficial to plant perception, and they respond to diverse environmental stressors, such as osmotic stress and high temperature [59–61]. In our study, the expression of Hsp90 (Unigene40313_All, 1.78-fold), CDPK (Unigene26962_All, 1.35-fold) and EDS1 (gi|158,282,407|gb|EDP08159_1|, 1.4-fold) was up-regulated in *U. prolifera* under the high-temperature stimulus. In addition, CDPK can activate H_2_O_2_ accumulation through regulating NADPH oxidase. The results indicated that the high-temperature stimulus accelerated ROS signalling, CDPK and EDS1 expression. In conclusion, the Ca^2+^-signal also played an important role in high-temperature stress in *U. prolifera*.

### Expression analysis of DEPs involved in other types of metabolisms

Linoleic acid (LA), a carboxylic acid, belongs to one of the two families of essential fatty acids. It is converted by various lipoxygenases and cyclooxygenases and the cytochrome P450 enzyme. All of these LA products exhibit bioactivity, and they are implicated in human physiology and pathology as indicated in the cited linkages. In the present study, 4 DEPs were mapped to linoleic acid metabolism. The expression of the alpha-beta hydrolase superfamily (gi|158279131|gb|EDP04893_1|, 2.05-fold), cytochrome P450 (Unigene18531_All, 1.23-fold), lipoxygenase (Unigene17329_All, 3.21-fold), and 12-oxophytodienoic acid reductase (CL2323_Contig1_All, 1.47-fold) was up-regulated. In addition, glycan, taurine, and hypotaurine played a key role in plant response to stress. The expression of glycan-biosynthesis-related proteins changed significantly under high-temperature stimulus. The expression of dolichyl-phosphate beta-glucosyl transferase (ALG_5_, Unigene5913_All, 1.29-fold) was up-regulated. However, alpha-1,2-mannosyltransferase (ALG_9_, CL1555_Contig1_All, 0.78-fold) was down-regulated. Glycosyl transferase is encoded by two DEPs; one is down-regulated, and the other is up-regulated. Moreover, it has been suggested that taurine, hypotaurine and their metabolic precursors (cysteic acid, cysteamine and cysteine sulphinic acid) might act as antioxidants through scavenging ·OH, HOCl and H_2_O_2_. It is concluded that cysteamine and hypotaurine are far more likely to act as antioxidants in vivo than taurine, provided that they are present in a sufficient concentration at sites of oxidant generation [62]. In our study, the expression of cystathionine beta-synthase (Unigene42486_All, 1.47-fold) was observed to be up-regulated. This result indicates that the high-temperature stimulus induced cysteamine biosynthesis and increased the antioxidant levels.

### qPCR to verify the reliability of the proteome

The expression of 8 unigenes were investigated via qPCR. The results were consistent with the available proteome data (Additional file 1: Table S1). Quantitative real time polymerase chain reaction results indicated that the unigenes that encode heat shock protein 90, APX, MnSOD, glutathione reductase, glutathione S-transferase, RbcL, heat shock protein 70 and ATP synthase were all significantly up-regulated by 3.152, 2.502, 2.106, 0.420, 2.320, 2.522, 2.242 and 3.252-fold, respectively.

### Validation of differentially expressed proteins identified by western blot

**Fig. 11:**
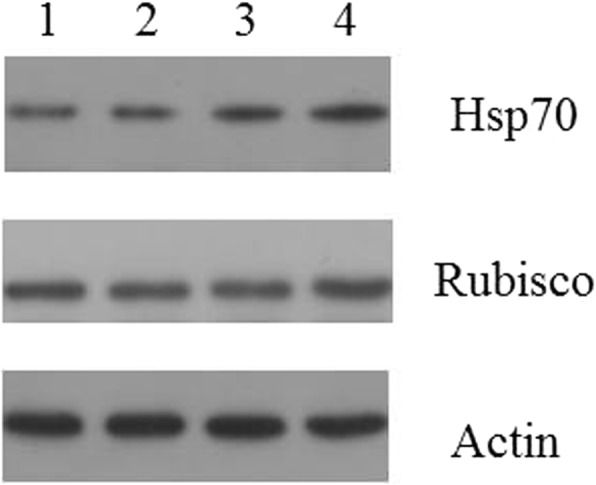
Western blotting.showing the changes of Hsp70 and RbcL level in UpHT compare with the control UpC. Note: 1.UpC(Control), 2. UpC(Control), 3.UpHT (High temperature), 4. UpHT (High temperature)

Two proteins, Hsp70 and RbcL identified DEPs with marked differences in expression determined by iTRAQ based quantitative analysis were selected to be verified by western blot analysis (Fig. 11). Hsp70 and RbcL protein was significantly up-regulated in UpHT groups as compared with control group (*p* < 0.05). The results which were found by western blot is consistent with the findings in iTRAQ analysis. It is well established that Hsp70 and RbcL could play a role in protecting the protein structure and photosynthesis early in the stress response.

### PPI network

**Fig. 12:**
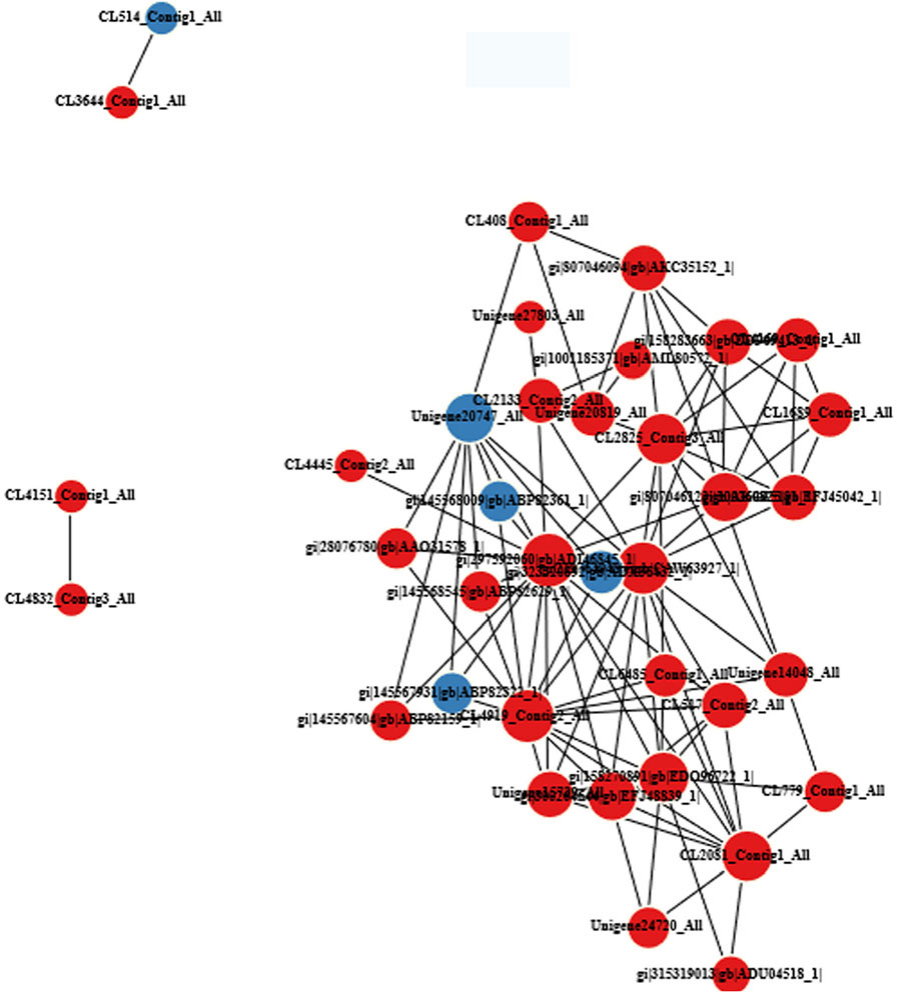
PPI network of *U. prolifera* response to high temperature stimuls. Red indicated the expression of protein up-regulated,blue indicated the expression of protein down-regulated. CL2081_Contig1_All: heat shock protein 90C; gi|158270891|gb|EDO96722_1|:heat shock protein 90C; gi|219764948|emb|CAW63927_1|:heat-shock inducible Hsp70;Unigene20819_All:F1F0 ATP synthase gamma subunit; CL517_Contig2_All:ClpB chaperone, Hsp100 family; gi|1001185371|gb|AML80572_1|:NADH-ubiquinone oxidoreductase 49 kDa subunit; CL4445_Contig2_All:prohibitin; gi|315319013|gb|ADU04518_1|:LhcSR; gi|300264644|gb|EFJ48839_1|:Stress-induced-phosphoprotein; CL408_Contig1_All:soluble inorganic pyrophosphatase 2; gi|300257667|gb|EFJ41912_1|:Serine/threonine-protein kinase CTR1;CL2825_Contig3_All:component of cytosolic 80S ribosome and 40S small subunit;Unigene15739_All:ClpB chaperone, Hsp100 family; CL4169_Contig1_All:nucleolar protein, component of C/D snoRNPs; Unigene20747_All:plastid transketolase; Unigene24720_All:DNA binding helix-turn helix protein; CL2133_Contig2_All:iron-sulfur subunit of mitochondrial succinate dehydrogenase;gi|297,592,060|gb|ADI46845_1|:heat shock protein 70B;CL4919_Contig2_All:flagellar associated protein;gi|300260825|gb|EFJ45042_1|:component of cytosolic 80S ribosome and 60S large subunit; CL7329_Contig1_All:histidine-aspartic acid phosphotransferase 1;CL4832_Contig3_All:ATP-dependent Clp protease regulatory subunit ClpC

In plants, proteins do not function in isolation within cells but as part of a network. In this study, a PPI network was generated to highlight the interactions and relationships between different proteins. To obtain interaction between proteins, we constructed a PPI network that was based on data downloaded from the STRING database. For the UpHT vs UpC protein, we chose 102 proteins that were involved in response to stimulus, photosynthesis, carbon fixation in photosynthetic organisms, oxidative phosphorylation, glutathione metabolism, plant signal transduction and plant-pathogen interaction to construct the PPI. Based on the differential expression patterns at the protein levels in UpHT compared to UpC (Fig. 12). In the present study, there are 37 unigenes nodes and 194 interactions in the PPI network. The PPI network indicated that heat shock protein 90C (CL2081_Contig1_All, 1.49-fold), heat shock protein 90C (gi|158270891|gb|EDO96722_1|, 1.44-fold), heat shock protein 70 (gi|219764948|emb|CAW63927_1|, 1.4-fold), ClpB chaperone, Hsp100 family (CL517_Contig2_All and Unigene15739_All, 1.64-fold and 1.46-fold) and heat shock protein 70B (gi|297592060|gb|ADI46845_1|, 1.82-fold) are involved in interactions with proteins from other functional groups and played important signal regulated. In addition, soluble inorganic pyrophosphatase 2 (CL408_Contig1_All, 1.51-fold), plastid transketolase (Unigene20747_All, 0.43-fold), F1F0 ATP synthase gamma subunit (Unigene20819_All, 1.69-fold), ribulose-1,5-bisphosphate carboxylase/oxygenase large subunit (gi|323,320,892|gb|ADX36432_1|, 0.8-fold), serine/threonine-protein kinase CTR1 (gi|300257667|gb|EFJ41912_1|, 2.66-fold), component of cytosolic 80S ribosome and 40S small subunit (CL2825_Contig3_All, 1.3-fold), flagellar associated protein (CL4919_Contig2_All, 1.53) as the key regulation factor and construct PPI under high temperature.

### A pathway model of high temperature stress responses in *U. prolifera*

**Fig. 13:**
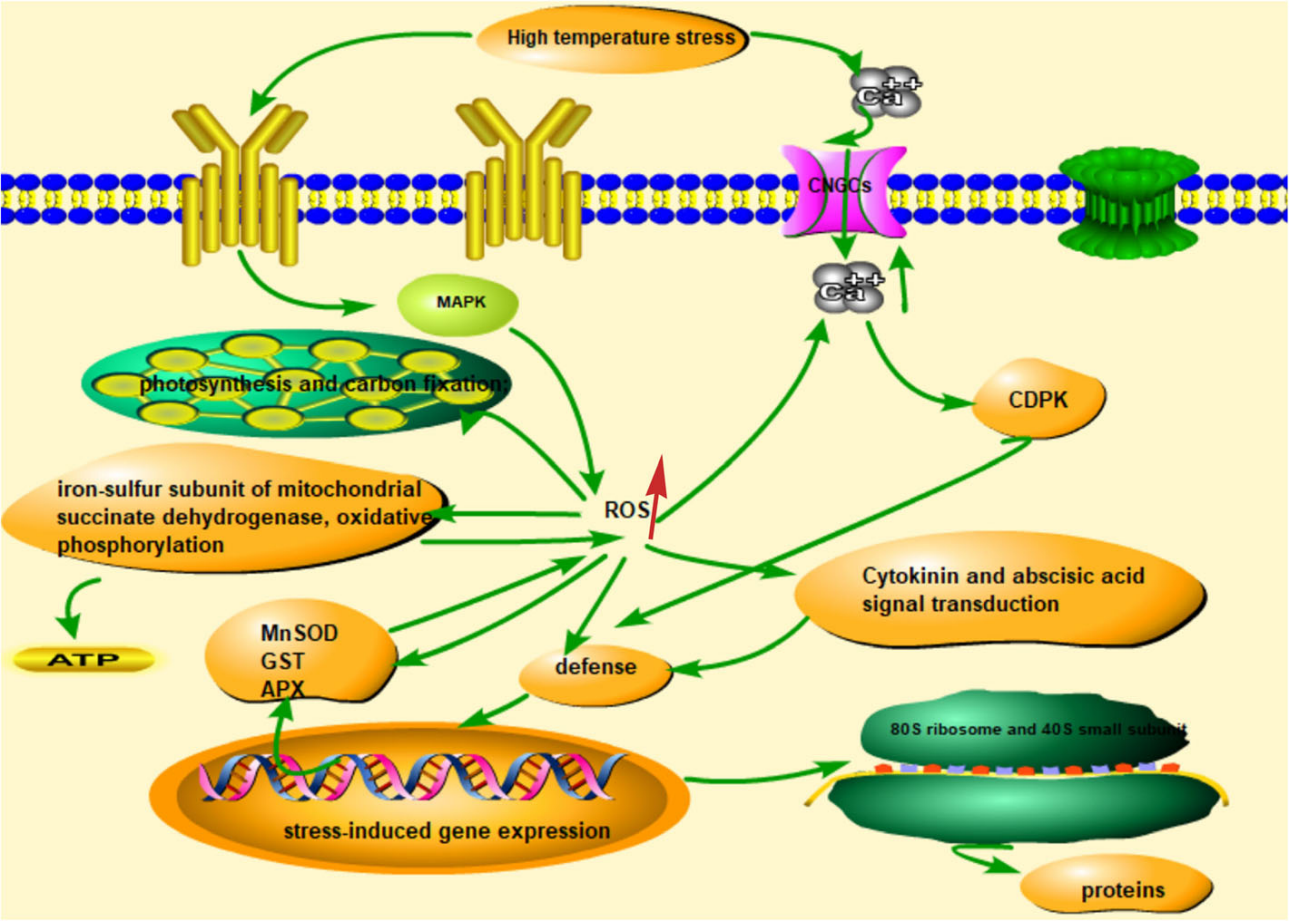
A pathway model of high temperature stress responses in *U. prolifera.* MAPK: mitogen-activated protein kinase; CNGC:cyclic-nucleotide gated channel;CDPK:Calcium-dependent protein kinases;ROS: reactive oxygen species;APX: ascorbate peroxidase;GST:glutathione S-transferase;MnSOD: manganese superoxide dismutase

Using our results and previous studies, we propose a putative synergistic response network for *U. prolifera* that responds to high-temperature stress. As shown in Fig. 13, the reaction of *U. prolifera* to high-temperature stress rapidly accumulates ROS, which leads to a series of metabolic changes. First, high-temperature stress induces stress signals to generate differential accumulation of signal transduction components. High temperature causes the cell ROS concentration to rapidly increase, and high concentrations of ROS result in an increasing release of Ca^2+^ into the cytoplasm. When the concentration of Ca^2+^ reaches a particular threshold, CDPK is activated. As a result, plants are able to produce appropriate responses to the stress conditions. In an emergency response, HSP, as the key molecule, is significantly up-regulated to protect proteins from misfolding. To remove the accumulated ROS, the antioxidant system is activated, and proteins that regulate the redox balance, including MnSOD, GSTs, APX and other response factors, are up-regulated. These proteins can break down excessive intracellular ROS and restore the normal intracellular redox environment. Proteins with important functions in energy metabolism are continuously upregulated to generate ATP, which allows plants to perform vital metabolic functions and to combat stress. Oxidative phosphorylation is the major approach to generate ATP, accompanied by the accumulation of ROS. The high-temperature stimulus induced upregulation of electron transport chain complex protein: NADH:ubiquinone oxidoreductase, succinate dehydrogenase, cytochrome C oxidase subunit, and ATP synthesis subunit in mitochondria. The imbalance between the activity changes of ETC may lead to accumulation of reactive oxygen species. The possible reason was that changes in the quinone pool redox state are responsible for the apparent inorganic phosphate activation of complex III. Complex III is responsible for higher ROS production during physiological working conditions relative to complex I [63]. At the same time, protein synthesis increased, and cytosolic 80S ribosome and 40S small subunit, nucleolar protein, and component of C/D snoRNPs were all up-regulated. However, the effect of high temperature on proteins involved in photosynthesis and carbon fixation are downregulated, and the result was consistent with the proteome response of Brachypodium distachyon to drought stress [64].

### Biological index and photosynthesis index

Antioxidant enzymes played an important role in preventing the oxidation of biological molecules and protecting plant tissues from damage caused by ROS that was induced by multiple environmental stresses. SODs catalysed O^2−^ + 2H^+^ → O_2_ + H_2_O_2_. In addition, APX and CAT catalysed H_2_O_2_ → O_2_ + H_2_O. In the present study, the effects of high temperature were investigated on the activity of antioxidant enzymes, the chlorophyll content, and the chlorophyll fluorescence parameter of *U. prolifera*. The result indicated that under a high-temperature stimulus, the protein content decreased during the all process compared to the control group (Additional file 1: Figure S1). SOD activity was decreased under high temperature compared to the control group. However, the APX activity increased in the later period (Additional file 1: Figure **S2**, Fig. 4). The MDA content increased over the time and reached the highest value after 96 h, which was significantly different from the control group (Additional file 1: Figure S3). Furthermore, the value of Fv/Fm dropped to the lowest point after 96 h of culture (Additional file 1: Figure S5). Our results provide important information for the selection of heat-resistant *U. prolifera*.

## Conclusions

A total of 1223 differentially expressed proteins were identified in response to heat stress. The up-regulated proteins were enriched in ribosome, oxidative phosphorylation, proteasome, glutathione metabolism, SNARE interactions in vesicular transport and linoleic acid metabolism pathway. The down-regulated proteins were in photosynthesis, carbon fixation, photosynthesis antenna proteins, and metabolic pathways. The up-regulated proteins were mainly glutathione S-transferases, heat shock proteins, manganese superoxide dismutase, ubiquitin-related protein, lhcSR, rubisco activase, serine/threonine protein kinase 2, adenylate kinase, Ca^2+^-dependent protein kinase (CDPK), disease resistance protein EDS1, mov34-domain-containing protein, metacaspase type II, NDPK2a, 26S proteasome regulatory subunit, ubiquinone oxidoreductase, ATP synthase subunit and cytochrome C oxidase subunit AHP, SnRK2s, and cytochrome P450. The down-regulated proteins were photosynthesis-related proteins, glutathione reductase, thioredoxin, thioredoxin peroxidase and carbon-fixation-related proteins. Overall, stress response, oxidative phosphorylation, cytokinin signal transduction pathway, abscisic acid signal transduction pathway, and Ca^2+^ signal transduction played key roles in high-temperature regulation. Moreover, high temperature might lead to photosynthesis and inhibition of carbon fixation in *U. prolifera.* These novel proteins provide a good starting point for further research into their functions using genetic or other approaches. These findings significantly improve the understanding of the molecular mechanisms that are involved in the tolerance of algae to high-temperature stress.

## Additional files

Additional file 1: **Figure S1.** Effects of high temperature on protein content of *U. prolifera*. **Figure S2.** Effects of high temperature on superoxide dismutase activity of *U. prolifera*. **Figure S3.** Effects of high temperature on MDA contents of *U. prolifera*. **Figure S4.** Effects of high temperature on APX activity of *U. prolifera*. **Figure S5.** Effects of high temperature on the maximum quantum yield of *U. prolifera*. **Table S1.** qPCR validation of the proteome data. (DOCX 402 kb)
