# Supplementary material for: Comparative proteomic analysis of Ulva prolifera response to high temperature stress
Source: Proteome Sci. 2018 Oct 27;16:17. doi: 10.1186/s12953-018-0145-5 (PMC6204280; doi:10.1186/s12953-018-0145-5)
Supplement: Supplementary file 1 — Figure S1. Effects of high temperature on protein content of U. prolifera. Figure S2. Effects of high temperature on superoxide dismutase activity of U. prolifera. Figure S3. Effects of high temperature on MDA contents of U. prolifera. Figure S4. Effects of high temperature on APX activity of U. prolifera. Figure S5. Effects of high temperature on the maximum quantum yield of U. prolifera. Table S1. qPCR validation of the proteome data. (DOCX 402 kb) [file 12953_2018_145_MOESM1_ESM.docx]

Comparative proteomic analysis of the response of *Ulva prolifera* to high-temperature stress

Meihua Fan^1^**^＊^**, Xue Sun^2^, Zhi Liao^1^, JianxinWang^1^, Yahe Li^2^, Nianjun Xu^2^**^＊^**

**Additional file 1: Figures and Tables**


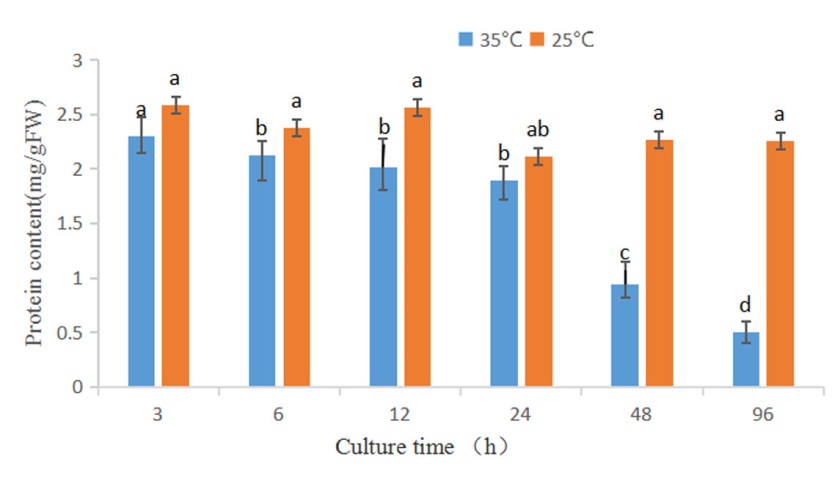


**Figure S1 Effects of high temperature on protein content of *U. prolifera***


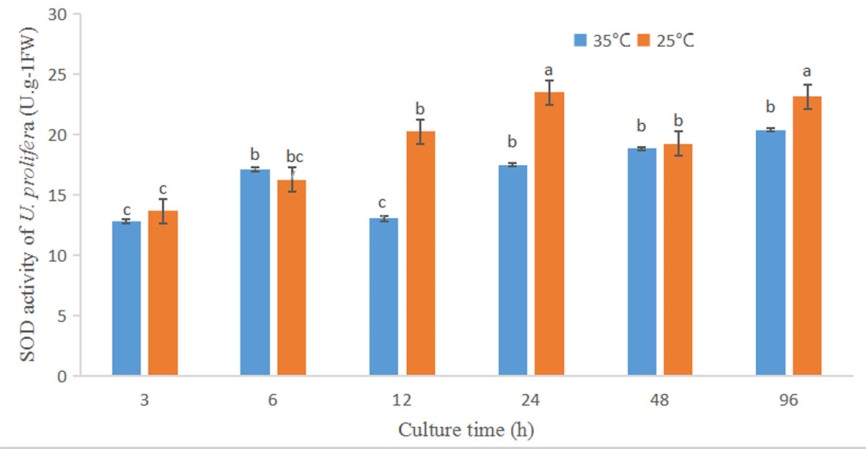


**Figure S2 Effects of high temperature on superoxide dismutase activity of *U. prolifera***


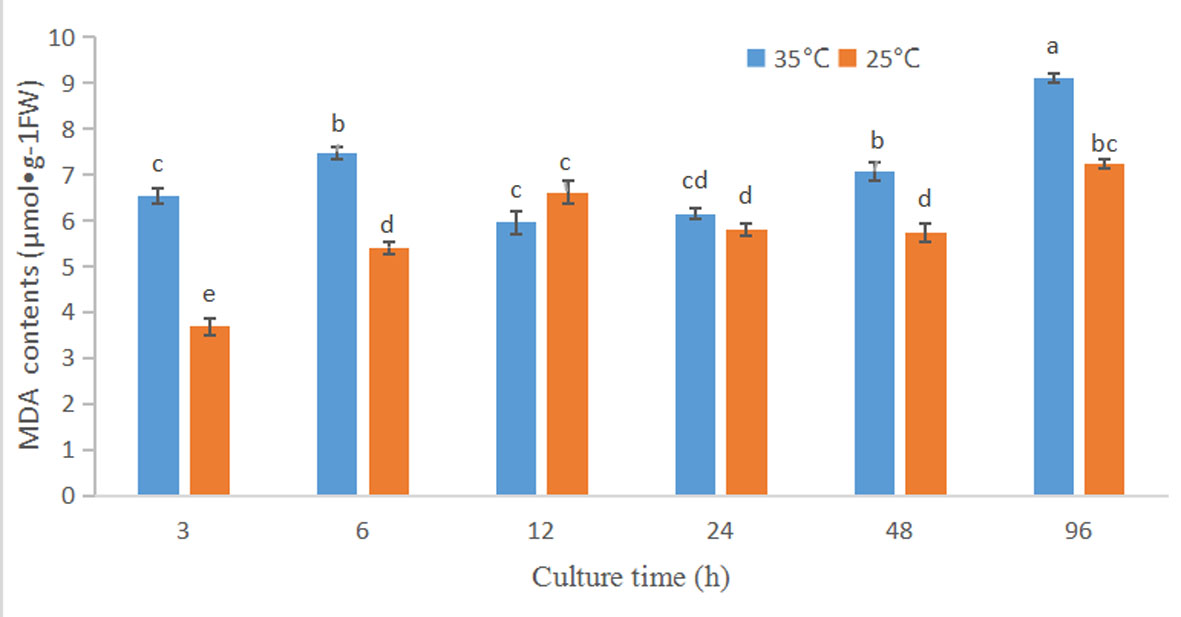


**Figure S3 Effects of high temperature on MDA contents of *U. prolifera***


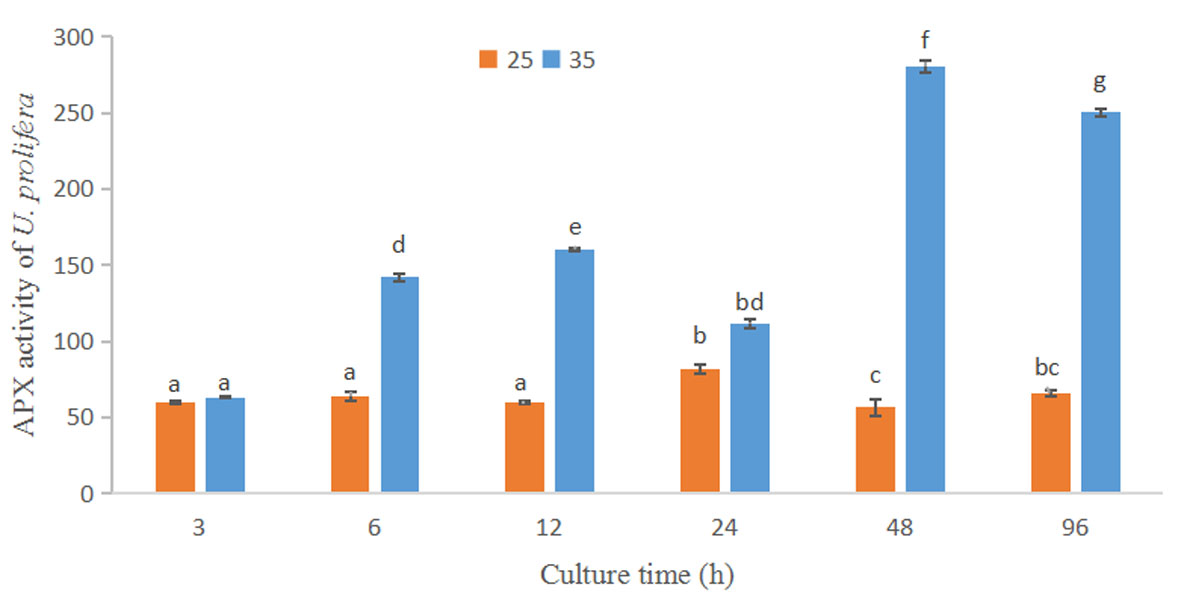


**Figure S4 Effects of high temperature on APX activity of *U. prolifera***


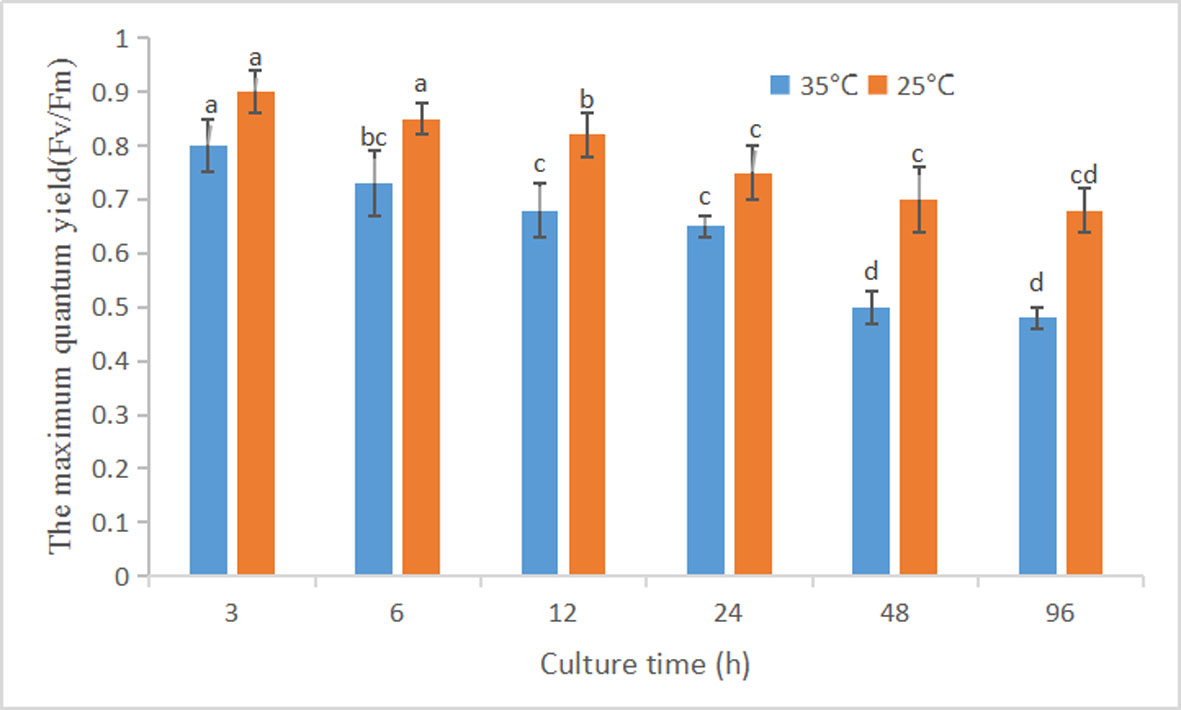


**Figure S5 Effects of high temperature on the maximum quantum yield of *U. prolifera***

**Table S1: qPCR validation of the proteome data**

| Gene ID | Annotation | Primer | Primer sequence | | Mean ratio (UpHT vs UpC) | qPCR (2^-ΔΔct^) |
| --- | --- | --- | --- | --- | --- | --- |
| CL5666.Contig2_All | Heat shock protein 90 | HSP90-F | | GTTCTATGAGGCATTCGG | 1.5±0.152 | 3.152±0.006 |
|  |  | HSP90-R | | AGCTGTTGCACTGCGTAC |  |  |
| CL1454_Contig2_All | APX | APX-F | | AGTGACGGAGGAAGGAGAAAT | 1.28±0.014 | 2.502±0.014 |
|  |  | APX-R | | AATCCAACACGTTAAGCTCG |  |  |
| CL6691_Contig2_All | MnSOD | SOD-F | | ATTCCAGATGGCTTTCAG | 1.42±0.004 | 2.106±0.009 |
|  |  | SOD-R | | CCAGGATACAGTGGCTCA |  |  |
| CL514_Contig1_All | glutathione reductase | GR-F | | AGTGGTGGTGTAAGAGCAA | 0.77±0.001 | 0.420±0.012 |
|  |  | GR-R | | TGGACCCGTAAACAAATAG |  |  |
| Unigene15839_All | glutathione S-transferase | GST-F | | CAGATACAGCGGGTACGACC | 1.45±0.032 | 2.320±0.023 |
|  |  | GST-R | | ACGATTTCAACCGCTCACCT |  |  |
| gi\|145567604\|gb\|ABP82159_1\| | RbcL | RbcL-F | | CAGGTGGTATTCACGTTTGGC | 1.28±0.094 | 2.522±0.06 |
|  |  | RbcL-R | | CCTGGAGCATTACCCCAAGG |  |  |
| Unigene26892_All | Heat shock protein 70 | HSP70-F | | CAAGGCCACTGTGACCAA | 1.58±0.015 | 2.242±0.015 |
|  |  | HSP70-R | | ACCACCAGCACCACCATA |  |  |
| CL1185_Contig1_All | Vacuolar ATP synthase | ATPsynthase-F1 | | TGGAGCCGTATTGCACATGT | 1.61±0.008 | 3.252±0.006 |
|  |  | ATPsynthase-R1 | | CCTGAAGCTTCACGAGGTGT |  |  |
|  |  | β-Actin-F | | AGGATGCATACGTTGGTGAA |  |  |
|  |  | β-Actin-R | | TTGTGGTGCCAAATCTTCTC |  |  |
